# Molecular Hydrogen and the Uremic Skeleton: A Critical Review and Turnover-State-Dependent Redox Hypothesis in CKD-MBD

**DOI:** 10.3390/antiox15091137

**Published:** 2026-09-08

**Authors:** Po-Jen Hsiao, Ching-Tsai Hsu, Wen-Fang Chiang, Jenq-Shyong Chan, Li-Yen Huang, Chung-Chi Yang, Kuo-Cheng Lu

**Affiliations:** 1 Division of Nephrology, Department of Internal Medicine, Taoyuan Armed Forces General Hospital, Taoyuan 325208, Taiwan; 2 Division of Nephrology, Department of Internal Medicine, Tri-Service General Hospital, National Defense Medical University, Taipei 11490, Taiwan; 3 Department of Life Sciences, National Central University, Taoyuan 320317, Taiwan; 4 Division of Cardiovascular Medicine, Taoyuan Armed Forces General Hospital, Taoyuan 325208, Taiwan; 5 Cardiovascular Division, Tri-Service General Hospital, National Defense Medical University, Taipei 11490, Taiwan; 6 School of Medicine, National Tsing Hua University, Hsinchu 300044, Taiwan; 7 Institute of Bioinformatics and Structural Biology, National Tsing Hua University, Hsinchu 300044, Taiwan; 8 Division of Nephrology, Department of Medicine, Taipei Tzu Chi Hospital, Buddhist Tzu Chi Medical Foundation, New Taipei City 23143, Taiwan; 9 Division of Nephrology, Department of Internal Medicine, Fu Jen Catholic University Hospital, New Taipei City 24352, Taiwan

**Keywords:** CKD-MBD, molecular hydrogen, osteoblast, osteoclast, redox signalling

## Abstract

Chronic kidney disease–mineral and bone disorder (CKD-MBD) confers a substantial fracture burden that is only partly addressed by therapies targeting phosphate, parathyroid hormone, and vitamin D. Redox dysregulation may represent a complementary mechanism: reactive oxygen species (ROS) are required for receptor activator of nuclear factor-κB ligand (RANKL)-dependent osteoclastogenesis, whereas excessive ROS impair Wnt/β-catenin signalling in osteoblast precursors and promote osteocyte dysfunction. Uremic toxins, inflammation, and dialysis further increase oxidative stress. Molecular hydrogen (H_2_) is a highly diffusible redox modulator that has been proposed to limit damaging radical-chain reactions while preserving physiological oxidant signalling. In non-uremic skeletal models, H_2_ consistently suppresses osteoclast differentiation and bone loss, but evidence for osteoblast rescue is heterogeneous. In CKD and dialysis, H_2_-based interventions have shown signals of reduced oxidative stress and symptomatic benefit; however, human evidence is predominantly observational, and no study identified in this review assessed a bone-specific endpoint. We therefore integrate uremic bone redox biology with H_2_ pharmacology and propose a turnover-state-dependent model in which H_2_ may restrain excessive resorption in high-turnover disease, while its net effect in low-turnover adynamic bone remains uncertain because potential osteoblast rescue competes with anti-osteoclastic activity established only in non-uremic models. H_2_ should therefore be considered an experimental, mechanistically differentiated strategy requiring direct evaluation in uremic models and turnover-stratified clinical trials with parallel skeletal and vascular safety endpoints.

## 1. Introduction

Chronic kidney disease (CKD) causes systemic disturbances in mineral metabolism, bone architecture, and extraskeletal calcification, collectively termed CKD-mineral and bone disorder (CKD-MBD); renal osteodystrophy (ROD) denotes its skeletal component [1,2,3]. Fracture risk rises as kidney function declines, and patients with end-stage kidney disease have a several-fold higher risk of hip fracture than age- and sex-matched individuals in the general population [4,5]. Fracture in this setting is associated with substantial morbidity and a two- to three-fold increase in subsequent mortality [4,6]. Even before dialysis, hip-fracture risk is elevated, while bone-density assessment and osteoporosis treatment remain underused [5,7].

Current management is centred on the mineral–hormonal axis, including phosphate control, active vitamin D analogues, calcimimetics, and selected antiresorptive or anabolic osteoporosis therapies [8,9,10]. These interventions often improve biochemical abnormalities more reliably than skeletal outcomes. An additional axis of injury is the redox environment in which bone cells differentiate, communicate, and mineralise [11,12].

Redox dysregulation is well characterised in CKD. Excess reactive oxygen and nitrogen species, combined with impaired antioxidant defences, promote lipid peroxidation, DNA and protein damage, inflammation, and fibrosis, while perturbing NRF2-KEAP1, NF-κB, hypoxia-inducible factor (HIF), and FOXO signalling [11,12,13,14]. The same pathways regulate skeletal cell fate. RANKL-induced osteoclastogenesis requires a transient TRAF6-Rac1-NADPH oxidase-dependent ROS burst [15,16], whereas oxidative stress redirects β-catenin from T-cell factor (TCF)-dependent transcription toward FoxO-dependent transcription in osteoblast precursors, thereby suppressing Wnt-driven osteoblastogenesis [17,18]. Increased skeletal ROS and impaired FoxO defence also contribute to age- and oestrogen-deficiency-associated bone loss [19,20,21]. Thus, bone is intrinsically redox-sensitive, and CKD imposes a chronic, multisource disturbance of that redox balance.

Attempts to translate redox biology into CKD therapy illustrate the difficulty of broadly manipulating oxidative signalling. Bardoxolone methyl, a potent NRF2 activator, increased estimated glomerular filtration rate in diabetic kidney disease, but was associated with fluid overload and cardiovascular safety concerns in BEACON [22]; subsequent programmes did not establish the anticipated hard-outcome benefit. Broad antioxidant supplementation has likewise shown inconsistent clinical translation. In bone, indiscriminate suppression of ROS is particularly problematic because physiological oxidant signalling is required for cell differentiation, and both insufficient and excessive NRF2 activity can disrupt skeletal homeostasis [23,24,25,26].

Molecular hydrogen (H_2_) differs conceptually from conventional antioxidant strategies. The original 2007 report proposed preferential reactivity with hydroxyl radical (•OH) and peroxynitrite (ONOO^−^) with relative sparing of superoxide, nitric oxide, and hydrogen peroxide [27,28]. Because H_2_ is electrically neutral, non-polar, and extremely small, it rapidly crosses biological membranes and can reach intracellular organelles, including mitochondria and nuclei [28,29]. Its direct molecular target remains debated, and current models extend beyond simple radical scavenging to include interruption of lipid free-radical chain reactions, modulation of oxidised phospholipid/Ca^2+^/NFAT signalling, indirect NRF2-KEAP1-ARE activation, suppression of NF-κB and MAPK pathways, and preservation of mitochondrial function [28,30,31,32,33].

In nephrology, H_2_ has been investigated mainly in dialysis-related redox modulation. Japanese studies of electrolysed H_2_-containing dialysis systems have reported feasibility, changes in oxidative-stress indices, haemodynamic and symptom signals, and improved composite outcomes in a prospective observational cohort [34,35,36,37,38]. A 2024 review further emphasised regulation of redox signalling and antioxidant gene expression, while noting practical limitations of inhaled H_2_ and the fluid burden associated with H_2_-rich water in CKD [13]. Oral solid formulations, including hydrogen-rich coral calcium (HRCC), may reduce these logistical constraints, although clinical data remain preliminary [13,39,40,41]. Separately, non-uremic skeletal studies indicate that H_2_ can suppress RANKL-induced osteoclast differentiation and preserve bone in several injury models, with less consistent evidence for osteoblast rescue [42,43,44,45,46,47,48,49,50]. No skeletal H_2_ study identified here used a uremic model, and no CKD H_2_ study reported bone-turnover, bone-density, histomorphometric, or fracture outcomes. The central question is therefore mechanistically plausible, but clinically untested.

This review addresses that gap by integrating two largely separate evidence bases: uremic skeletal redox biology and H_2_ pharmacology. We examine redox injury at the level of osteoclasts, osteoblast-lineage cells, osteocytes, and marrow stroma; we evaluate the chemistry, pharmacokinetics, and CKD-relevant delivery routes of H_2_; we distinguish established findings from single-model observations and mechanistic hypotheses; we critically appraise the human CKD/dialysis literature; and we define a staged translational programme. The intended conclusion is deliberately limited: H_2_ is a biologically plausible adjunctive redox strategy that warrants direct testing in renal bone disease, not an established therapy. This review is distinct from our earlier synthesis of H_2_ and redox signalling in the kidney [13]: its novel contribution is the integration of uremic skeletal redox biology with H_2_ pharmacology across osteoclasts, the osteoblast lineage, osteocytes, and marrow stroma; the formulation of a falsifiable turnover-state-dependent (bidirectional remodelling–normaliser) hypothesis whose defining test is a prespecified H_2_ × turnover-state interaction; and the definition of a staged, biomarker-anchored translational programme with parallel skeletal and vascular endpoints.

## 2. Methods: Scope, Search Strategy and Evidence Appraisal

This narrative critical review was not registered with PROSPERO and did not use duplicate independent screening or a formal risk-of-bias tool; it is therefore reported as a structured narrative review, rather than a PRISMA systematic review (see Section 11). We searched PubMed/MEDLINE, Web of Science Core Collection, and Embase from inception to 31 January 2026. To minimize omissions across adjacent evidence streams, PubMed was queried with deliberately broad, separate strategies for H_2_ + kidney and H_2_ + bone; the same dual logic—broad and combined H_2_ × CKD × skeletal strings—was then applied in Web of Science and Embase to harmonize strategies across databases. Full Boolean strings and the final search date are provided in Supplementary Table S2.

Eligibility followed the PICOTS framework (Supplementary Table S1). Included records addressed molecular hydrogen in adult chronic kidney disease, dialysis, uremic-toxin, skeletal, or bone-cell contexts and encompassed mechanistic/in vitro, animal, observational human, and interventional clinical studies. We restricted inclusion to adult populations and, for feasibility, to English-language publications; excluding non-English studies is acknowledged as managing a potential source of language bias.

Given the sparsity and heterogeneity of evidence—particularly the absence of any study simultaneously evaluating H_2_, CKD, and a skeletal endpoint—formal quantitative synthesis would be inappropriate. Accordingly, each statement in Section 5 is graded using three descriptive tiers applied consistently: (i) replicated preclinical evidence (concordant findings in ≥2 independent laboratories or models); (ii) single-study evidence (a single study or model); and (iii) mechanistic hypothesis (a biologically reasoned extrapolation not yet tested in bone). Human data are flagged as evidence of feasibility and biological activity, rather than efficacy. The complete lack of any skeletal H_2_ study in a uremic/CKD model is itself treated as a primary finding of this review.

## 3. The Uremic Skeleton as a Redox-Injured Organ

### 3.1. Why Bone Is Redox-Vulnerable in CKD

Bone is a continuously remodelling, metabolically demanding tissue in which osteoblasts, osteoclasts, and osteocytes must coordinate opposing functions within a shared microenvironment. Matrix-producing osteoblasts depend on mitochondrial energy metabolism; osteoclasts are macrophage-lineage cells whose differentiation and resorptive function require oxidant signalling; and long-lived osteocytes coordinate remodelling through RANKL, osteoprotegerin (OPG), sclerostin, and FGF23 [21,51,52]. All three lineages use ROS as physiological signals, but are damaged when ROS become excessive. This dual requirement makes the skeleton particularly sensitive to the sustained shift in redox set-point imposed by CKD.

CKD-related skeletal fragility cannot be explained by turnover rate or areal bone mineral density alone. Fractures occur at BMD values that may not appear severely reduced, reflecting deterioration in bone quality, including collagen cross-linking, mineral crystallinity, cortical porosity, and microdamage [8,53,54]. Oxidative and carbonyl stress can alter collagen cross-links and carbonylate matrix proteins [53,54], providing a direct mechanistic link between the uremic redox milieu and impaired bone material properties. Redox biology may therefore help explain a component of fracture risk that is not captured by conventional mineral-axis markers.

### 3.2. Cellular and Enzymatic Sources of ROS in Bone

Four major enzymatic and organellar systems contribute to ROS generation in the skeletal microenvironment.

*NADPH oxidases.* NOX1, NOX2 and NOX4 are expressed in bone and marrow cells. NOX2, assembled with p22phox and cytosolic regulatory subunits, is the principal source of the superoxide burst that follows RANK ligation in osteoclast precursors; NOX4 resides additionally in mitochondrial, endoplasmic reticulum, and nuclear membranes [15,16,55]. In the kidney, NOX2 and NOX4 are the dominant renal isoforms, and their dysregulation is a recognised contributor to diabetic and hypertensive nephropathy [13,55,56].

*Mitochondria.* Electron leakage from respiratory-chain complexes I and III generates superoxide, which is converted by SOD1 and SOD2 to H_2_O_2_ and subsequently reduced by glutathione peroxidases and peroxiredoxins [13,57]. Mitochondrial dysfunction and impaired mitophagy have been implicated in abnormal bone remodelling in CKD-MBD [58]. Because matrix synthesis and mineralisation are energy-intensive, mitochondrial competence is directly relevant to osteoblast function.

*Xanthine oxidoreductase.* Conversion of xanthine dehydrogenase to xanthine oxidase in ischaemic or inflamed tissue generates superoxide and H_2_O_2_; xanthine oxidase inhibition has renoprotective and cardiorenal protective signals in CKD [13,59].

*Myeloperoxidase.* Released from neutrophil azurophilic granules, MPO generates hypochlorous acid; the MPO–H_2_O_2_–chloride system is a recognised pathophysiological contributor to experimental glomerular and tubulointerstitial disease, and MPO is elevated in dialysis populations as a marker of inflammatory oxidative burden [13,60].

### 3.3. Uremia-Specific Redox Drivers

Superimposed on these constitutive sources are drivers unique to, or greatly amplified in, kidney failure.

*Protein-bound uremic toxins.* Indoxyl sulfate (IS) and p-cresyl sulfate (pCS) accumulate as glomerular filtration declines and are inefficiently removed by conventional dialysis because of albumin binding. IS increases mitochondrial ROS, impairs osteoblast viability and differentiation, downregulates PTH1R, and interferes with Wnt signalling [61,62,63,64,65]. pCS impairs osteoblast function through JNK and p38 MAPK activation [66]. In osteocytes, IS increases sclerostin and DKK1 expression and alters the RANKL/OPG balance [65,67]. These findings link retained uremic solutes directly to redox-sensitive skeletal dysfunction.

*Hyperphosphataemia, SHPT, skeletal PTH resistance, and Klotho deficiency.* Phosphate retention promotes FGF23 and PTH elevation, while sustained secondary hyperparathyroidism (SHPT) increases RANKL and suppresses OPG, favouring high-turnover resorption [8,58,68]. At the same time, uremic toxins can blunt osteoblastic PTH responsiveness, which helps explain why a single PTH concentration does not reliably define bone turnover in CKD [8,61,68]. Renal α-Klotho declines early in CKD; beyond its role as an FGF23 co-receptor, Klotho has antioxidant and anti-apoptotic actions, and its deficiency contributes to phosphate retention, vascular calcification, cellular ageing, and impaired stress resistance [69,70,71]. Klotho therefore links the mineral and redox axes, and is a plausible biomarker for redox-directed interventions.

*Metabolic acidosis and inflammation.* Chronic acidosis promotes mineral dissolution and favours osteoclastic resorption. Uremia-associated inflammation, driven by retained toxins, gut dysbiosis, dialysis-related factors, and vascular access, sustains cytokines such as IL-1β, IL-6, TNF-α, and MCP-1, which promote RANKL-dependent resorption and suppress osteoblastogenesis [58,72].

*Dialysis-related oxidative stress.* Haemodialysis can acutely increase oxidative burden through blood–membrane contact, leukocyte activation, and loss of low-molecular-weight antioxidants. Dissolved-H_2_ dialysis approaches have been associated with changes in albumin redox indices in dialysis settings, while high-H_2_ dialysate can also alter the interaction between albumin and indoxyl sulfate [35,73]. These observations support the broader premise that dialysis-based H_2_ delivery can modify systemic or dialysate redox-related processes, although they do not establish a skeletal effect.

### 3.4. Redox-Sensitive Transcriptional Nodes

**Figure 1:**
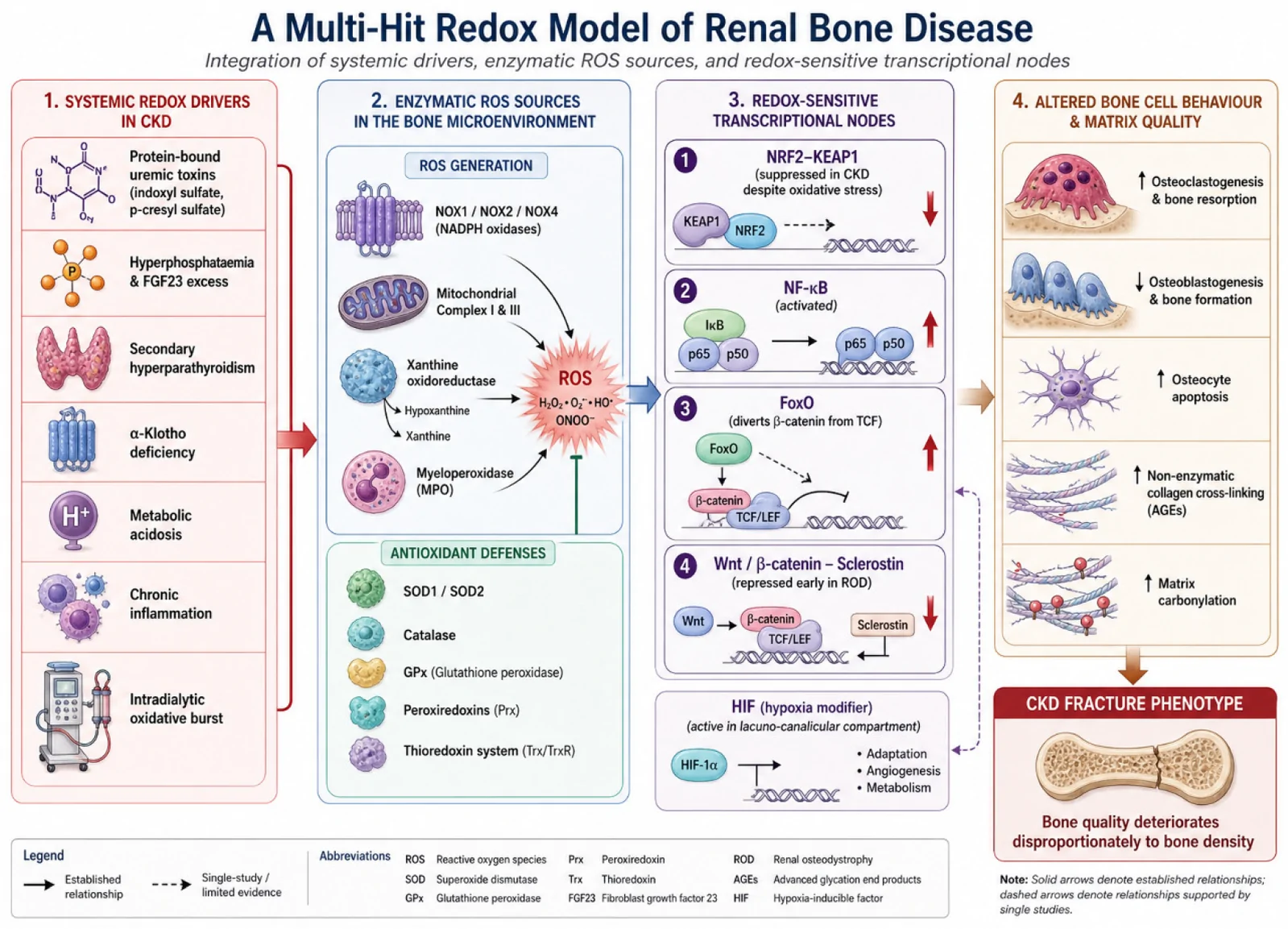
A multi-hit redox model of renal bone disease. Schematic integrating the four classes of redox driver operating on the uremic skeleton. Left panel: systemic drivers—protein-bound uremic toxins (indoxyl sulfate, *p*-cresyl sulfate), hyperphosphataemia and FGF23 excess, secondary hyperparathyroidism, α-Klotho deficiency, metabolic acidosis, chronic inflammation, and the intradialytic oxidative burst. *Centre panel:* enzymatic ROS sources within the bone microenvironment—NOX1/2/4, mitochondrial complexes I and III, xanthine oxidoreductase and myeloperoxidase—balanced against SOD1/2, catalase, GPx, peroxiredoxin and thioredoxin systems. Right panel: the four redox-sensitive transcriptional nodes through which these inputs are translated into altered bone-cell behaviour—NRF2–KEAP1 (suppressed in CKD, despite oxidative stress), NF-κB (activated), FoxO (diverting β-catenin from TCF), and Wnt/β-catenin–sclerostin (repressed early in ROD)—with HIF shown as a modifier of the hypoxic lacuno–canalicular compartment. Downstream outputs are indicated as increased osteoclastogenesis, impaired osteoblastogenesis, osteocyte apoptosis, non-enzymatic collagen cross-linking and matrix carbonylation, converging on the CKD fracture phenotype in which bone quality deteriorates disproportionately to bone density. Solid arrows denote established relationships; dashed arrows denote relationships supported by single studies.

Four redox-sensitive transcriptional nodes are particularly relevant to bone-cell responses in CKD and to the proposed actions of H_2_ (Figure 1).

*NRF2-KEAP1.* Under basal conditions, KEAP1 targets NRF2 for ubiquitin–proteasome degradation. Oxidative or electrophilic modification of KEAP1 cysteines permits NRF2 accumulation and ARE-dependent transcription of genes including HMOX1, NQO1, glutathione S-transferases, catalase, GPX1, and enzymes involved in glutathione synthesis [74,75]. Despite persistent oxidative stress, NRF2 activity may be impaired in CKD, and has been implicated in disease progression [13,76,77]. In bone, NRF2 generally restrains osteoclastogenesis, whereas excessive NRF2 activation can inhibit osteoblast differentiation [23,24,25,78,79].

*NF-κB.* Cytosolic H_2_O_2_ can activate IKK, promoting IκB degradation and NF-κB-dependent transcription of inflammatory mediators. Conversely, NF-κB DNA binding and IKK activity are themselves redox-sensitive [13,80]. Because canonical NF-κB signalling is also required for RANKL-induced osteoclast differentiation, this pathway links uremic inflammation directly to bone resorption.

*FOXO.* FoxO transcription factors induce antioxidant defence genes and can protect osteoblasts from oxidative injury, while restraining osteoclastogenesis. However, FoxO also competes with TCF for β-catenin; under oxidative stress, β-catenin is diverted from pro-osteoblastic Wnt/TCF signalling toward FoxO-dependent transcription [17,18,19,20,81,82].

*Wnt/β-catenin-sclerostin and HIF*. Osteocyte-derived sclerostin and DKK1 inhibit LRP5/6-dependent Wnt signalling, and repression of osteocytic Wnt/β-catenin activity is an early feature of progressive ROD [83]. Sclerostin concentrations increase in CKD, although its interpretation as a bone biomarker remains complex. HIF signalling is regulated by oxygen tension and prolyl hydroxylase activity, and contributes to adaptation within hypoxic bone and marrow compartments; HIF-prolyl-hydroxylase inhibitors are now used in renal anaemia, but their long-term skeletal consequences remain incompletely defined [13,84].

## 4. Molecular Hydrogen: Chemistry, Pharmacokinetics and Delivery

### 4.1. Physicochemical Basis of Tissue and Skeletal Access

**Figure 2:**
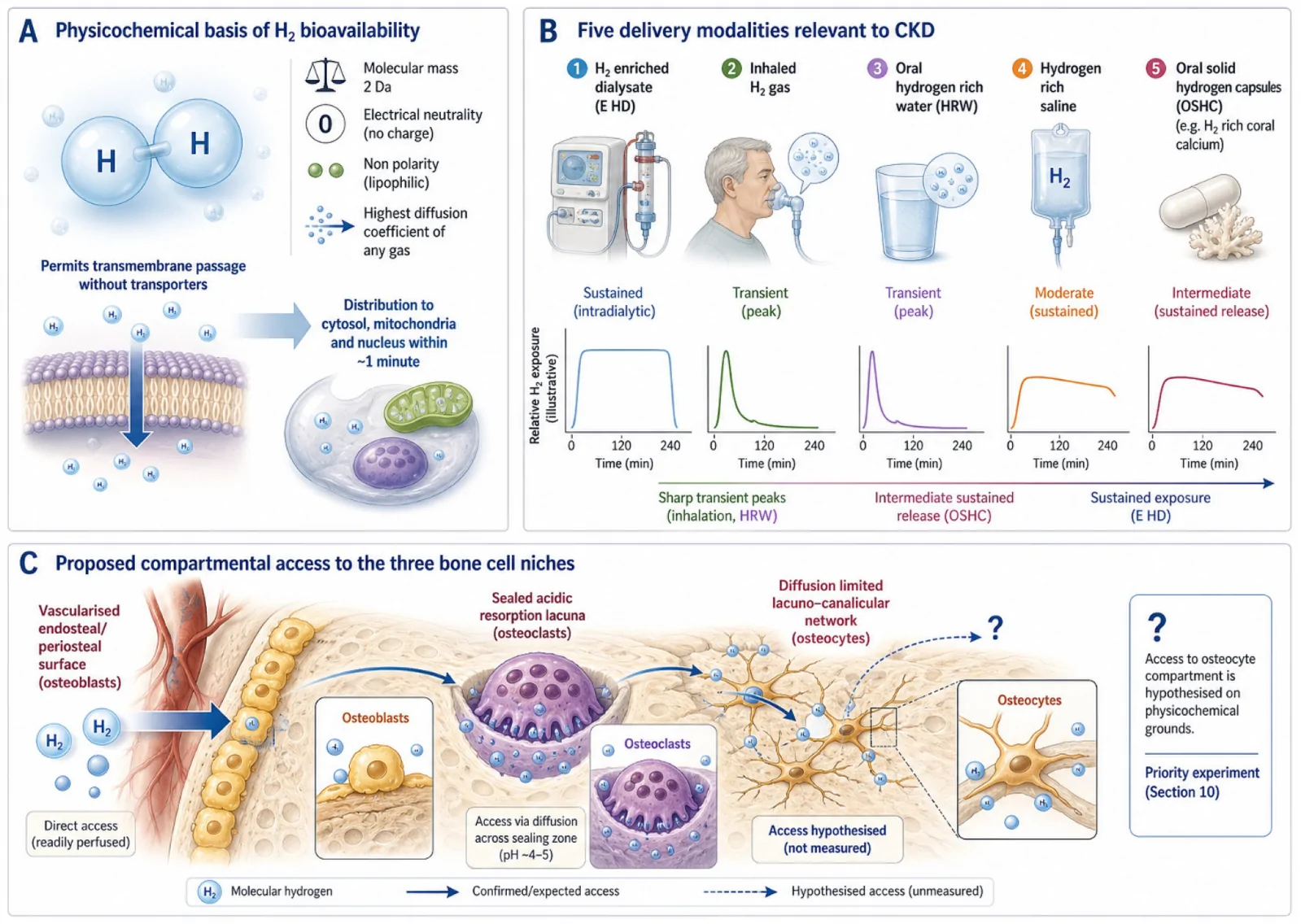
Molecular hydrogen: physicochemical properties, delivery modalities and compartmental access. Panel (**A**): physicochemical basis of H_2_ bioavailability—molecular mass 2 Da, electrical neutrality, non-polarity and high diffusivity, permitting transmembrane passage without transporters and distribution to cytosol, mitochondria and nucleus within approximately one minute. Panel (**B**): the five delivery modalities relevant to CKD—H_2_-enriched dialysate (E-HD), inhaled H_2_ gas, oral H_2_-enriched water, hydrogen-rich saline and oral solid hydrogen capsules including hydrogen-rich coral calcium—with schematic exposure–time curves illustrating sustained intradialytic exposure (E-HD), sharp transient peaks (HRW, inhalation) and intermediate sustained release (OSHC). Panel (**C**): proposed compartmental access to the three bone cell niches: the vascularised endosteal/periosteal surface (osteoblasts), the sealed acidic resorption lacuna (osteoclasts), and the diffusion-limited lacuno–canalicular network (osteocytes). Access to the osteocyte compartment is shown with a question mark: it is hypothesised on physicochemical grounds, has not been experimentally measured, and is identified in Section 10 as a priority experiment.

The physicochemical properties, delivery modalities, and proposed skeletal compartmental access of H_2_ are summarized in Figure 2. H_2_ is the smallest neutral molecule and has a high diffusion coefficient. These physicochemical properties allow rapid passage across biological membranes and access to intracellular compartments, including mitochondria and nuclei [13,28,29]. This broad diffusibility is relevant because redox signalling occurs within spatially restricted intracellular and tissue microdomains.

Potential skeletal access is, therefore, plausible but unproven. Osteocytes reside within a relatively hypoxic lacuno–canalicular network embedded in mineralised matrix, while osteoclasts create sealed acidic resorption lacunae. H_2_ could, theoretically, diffuse into each of these compartments; however, no study identified in this review directly measured H_2_ concentrations in bone, marrow, or the osteocyte lacuno–canalicular system. Skeletal pharmacokinetics should therefore be treated as an experimental priority, rather than an assumed advantage.

### 4.2. Direct Targets and the “Signal-Sparing Antioxidant” Concept

The original mechanistic model proposed that H_2_ preferentially reduces highly reactive species such as •OH and ONOO^−^ while relatively sparing superoxide, H_2_O_2_, and nitric oxide, which have physiological signalling roles [27]. This proposed signal-sparing property is central to the rationale for H_2_ in a tissue in which complete ROS suppression would be undesirable.

Direct radical scavenging alone is unlikely to explain the breadth of reported H_2_ effects at achievable tissue concentrations [13,28]. Additional models include interruption of lipid free-radical chain reactions and consequent changes in oxidised phospholipid/Ca^2+^-dependent transcription [30,31]; indirect modulation of NRF2-KEAP1 and antioxidant-response genes, potentially involving Wnt/β-catenin and GSK3β [13,32,85,86]; and preservation of mitochondrial function and quality-control programmes, including PGC-1α-associated pathways [33,87]. These mechanisms are complementary, rather than mutually exclusive.

The Ca^2+^/NFAT pathway is particularly relevant to bone. NFATc1 is the master transcriptional regulator of osteoclastogenesis, and is activated downstream of RANKL-induced Ca^2+^ oscillations and ROS signalling. H_2_ suppresses oxidised-phospholipid-driven Ca^2+^ signalling and NFAT activation in non-skeletal systems [30,31]. Whether the same mechanism contributes to H_2_-mediated inhibition of osteoclastogenesis has not been tested directly, and should be considered a specific, falsifiable hypothesis.

### 4.3. Delivery Modalities Relevant to CKD

Five delivery formats are relevant, compared in detail in Table 1.

*H_2_-enriched dialysate (E-HD).* Electrolysis-based systems generate dissolved H_2_ that is delivered across the dialyser during treatment, with concentrations of approximately 30–80 ppb reported in clinical systems [34,36,37]. This route is attractive in kidney failure because it uses an existing extracorporeal circuit, adds no oral fluid burden, and provides repeated systemic exposure during dialysis. High levels of dissolved H_2_ has also been reported to facilitate dissociation of indoxyl sulfate from albumin [73], raising the possibility of a combined redox and toxin-handling effect that requires in vivo confirmation. Experimental H_2_-containing peritoneal dialysis solutions have likewise shown protection of mesothelial and peritoneal membrane integrity [89].

*Inhaled H_2_ gas.* Inhaled H_2_ produces rapid systemic exposure and is useful for acute mechanistic studies, but chronic outpatient use in CKD is limited by equipment, supervision, adherence, and institutional safety requirements related to flammability [13,28,88].

*Oral H_2_-rich water (HRW).* Dissolved H_2_ in water can approach approximately 1.6 ppm at ambient pressure, but exposure is brief and highly dependent on preparation, container permeability, and time to ingestion. In dialysis populations, the fluid volume needed for repeated dosing is an important practical limitation because it conflicts with fluid restriction and interdialytic weight-management goals [13].

*H_2_-rich saline (HRS).* HRS permits controlled parenteral exposure and has been used extensively in preclinical studies, including several skeletal models [44,48,49]. Its value is primarily experimental because repeated parenteral administration and sodium loading are poorly suited to chronic CKD therapy.

*Oral solid hydrogen formulations, including HRCC.* Solid formulations generate H_2_ in the gastrointestinal tract and may provide longer exposure than HRW without an additional fluid load [13,39,40]. In a prospective four-week dose-ranging case series of 16 patients with metabolic syndrome, HRCC was tolerated across three dose levels and was associated with lower triglycerides, with no change in quality-of-life measures [39]. Preclinical work has also reported metabolic effects of coral-calcium-carried hydrogen [40]. These findings establish preliminary feasibility, rather than efficacy in CKD or bone disease.

For CKD-MBD, HRCC requires a specific safety qualification. A fluid-sparing solid formulation could be useful in non-dialysis CKD, but a calcium-carbonate carrier contributes to total calcium exposure. Calcium-based phosphate binders can promote positive calcium balance and are restricted in contemporary CKD-MBD management [8,9]. Any CKD trial of HRCC should therefore quantify elemental calcium per dose, account for concurrent dietary and binder calcium, and monitor serum calcium, PTH, and vascular calcification. Development of a non-calcium solid H_2_ carrier would avoid this confounding exposure, and should be prioritised.

## 5. Cell-Specific Molecular Actions of H_2_ in the CKD Skeleton

**Figure 3:**
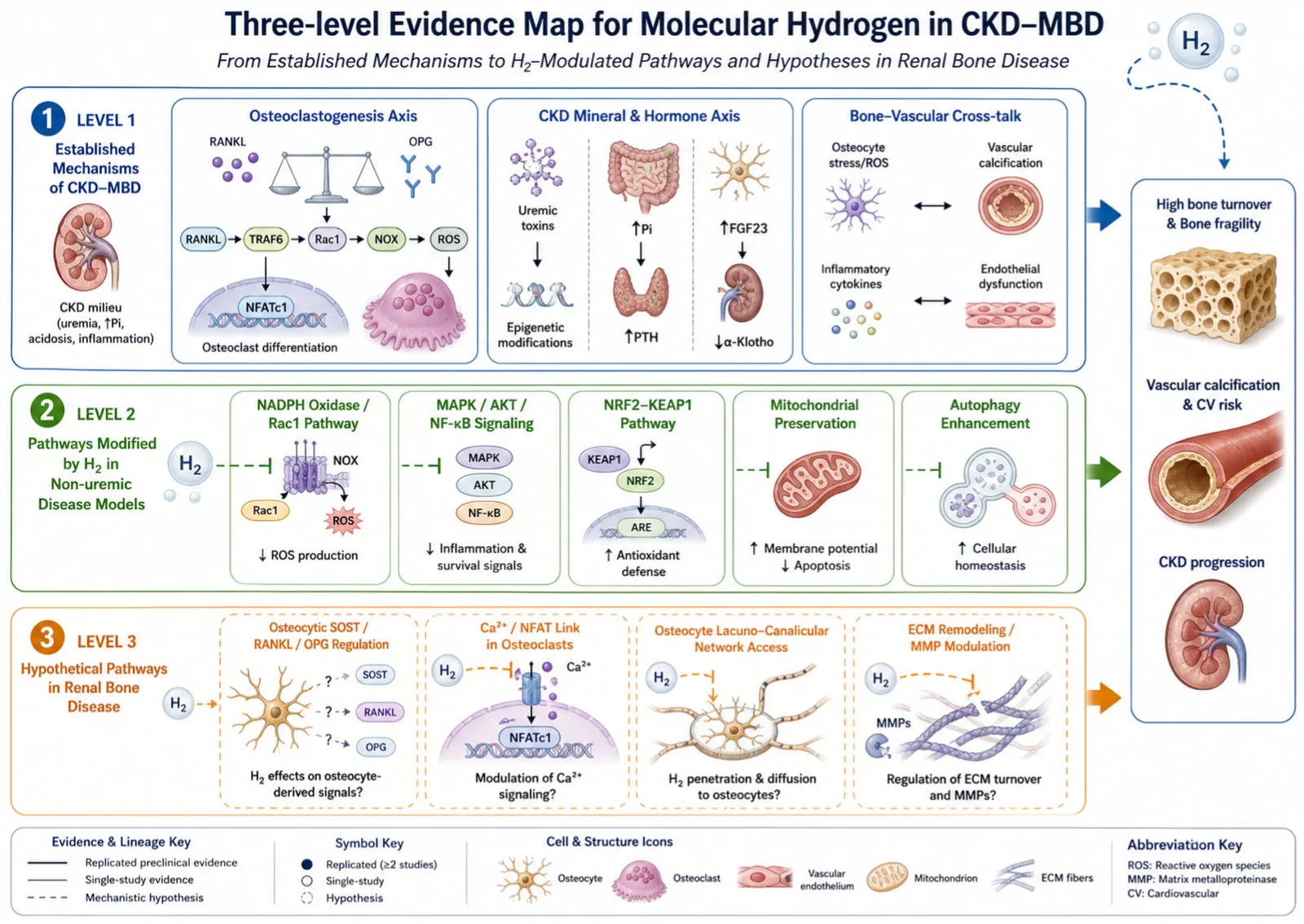
Three-level evidence map for molecular hydrogen in CKD-MBD. Each pathway invoked in this review is assigned to one of three evidence levels: (1) established mechanisms of CKD-MBD (e.g., RANKL/OPG and the RANKL–TRAF6–Rac1–NOX–ROS–NFATc1 axis; uremic-toxin, FGF23 and α-Klotho biology); (2) pathways demonstrated to be modified by H_2_ in non-uremic disease models (reduced NADPH-oxidase/Rac1 and MAPK/AKT/NF-κB signalling, NRF2–KEAP1 modulation, mitochondrial preservation); and (3) pathways that remain hypothetical in renal bone disease (H_2_ action on osteocytic SOST/RANKL/OPG, the Ca^2+^/NFAT link in osteoclasts, osteocyte lacuno–canalicular access, and ECM/MMP modulation). Solid symbols denote replicated preclinical evidence, thin symbols single-study evidence, and dashed symbols mechanistic hypotheses.

This section evaluates lineage-specific evidence using the evidence tiers defined in Section 2. A three-level evidence map summarizing the distinction between established CKD-MBD mechanisms, H_2_-modulated pathways demonstrated in non-uremic models, and mechanisms that remain hypothetical in renal bone disease is presented in Figure 3.

### 5.1. Osteoclasts: The RANKL–TRAF6–Rac1–NOX–ROS–NFATc1 Axis

**Figure 4:**
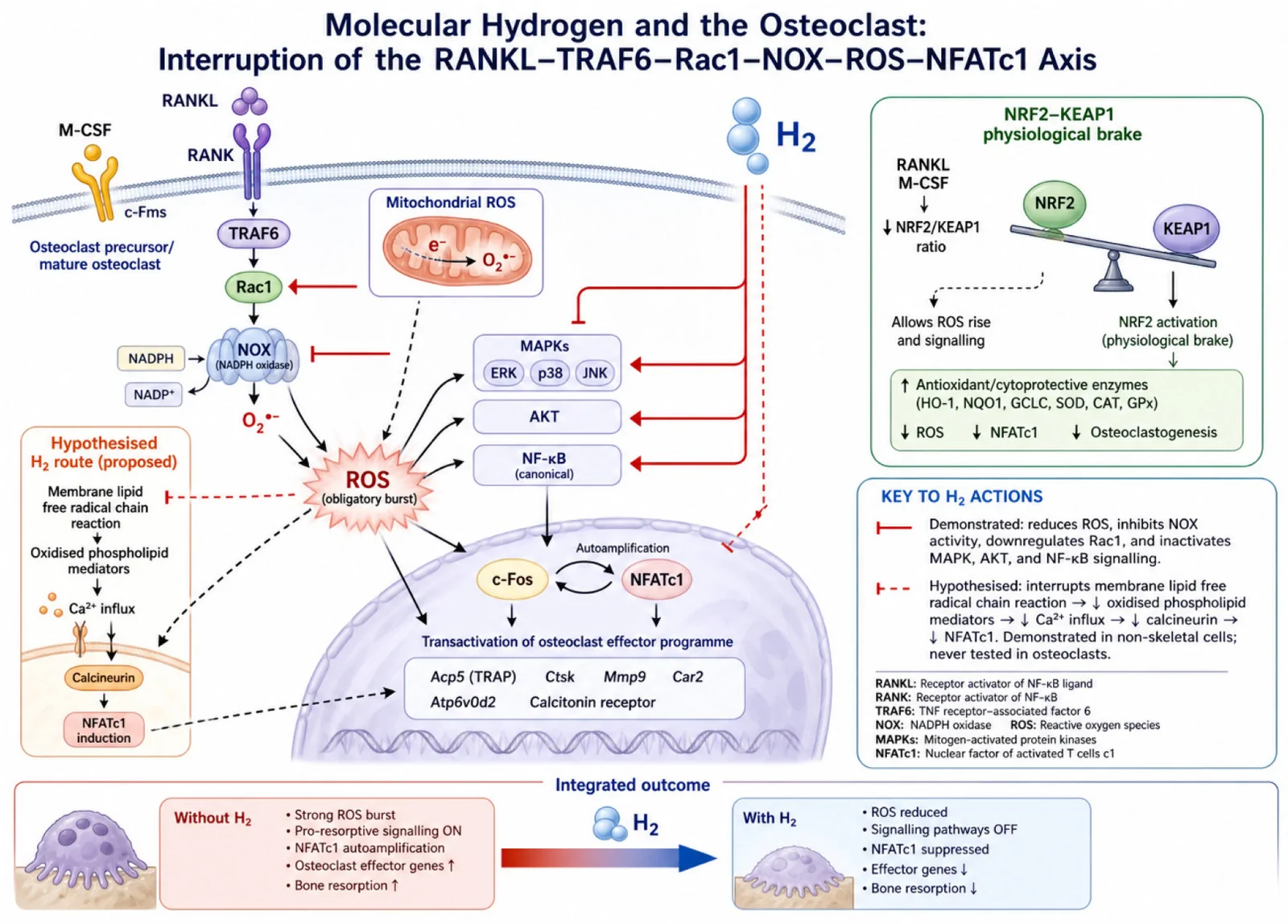
Molecular hydrogen and the osteoclast: interruption of the RANKL–TRAF6–Rac1–NOX–ROS–NFATc1 axis. RANKL binding to RANK recruits TRAF6, activating Rac1 and NOX-dependent superoxide generation, with parallel mitochondrial ROS contribution. The resulting obligatory ROS burst amplifies ERK, p38 and JNK MAPK signalling, AKT, and canonical NF-κB activation, converging on c-Fos and NFATc1 auto-amplification and transactivation of the osteoclast effector programme (*Acp5*/TRAP, *Ctsk*, *Mmp9*, *Car2*, *Atp6v0d2*, calcitonin receptor). NRF2 is shown as the physiological brake on this circuit: RANKL and M-CSF lower the NRF2/KEAP1 ratio to permit the ROS rise, while NRF2 activation restores cytoprotective enzyme expression and suppresses NFATc1. Demonstrated points of H_2_ action are marked with solid inhibition symbols—reduced intracellular ROS, suppressed NADPH oxidase activity, downregulated Rac1, and inactivated MAPK, AKT and NF-κB signalling. The hypothesised route is marked with a dashed inhibition symbol: H_2_-mediated interruption of the membrane lipid free-radical chain reaction reduces oxidised phospholipid mediators, attenuating Ca^2+^ influx, calcineurin activation and hence NFATc1 induction. This link has been demonstrated in non-skeletal cells but never tested in osteoclasts, and is proposed here as a unifying mechanism (Section 4.2, Section 10 priority iv).

The principal redox-sensitive pathways linking RANKL-driven osteoclastogenesis to the reported actions of H_2_ are summarized in Figure 4. Importantly, the H_2_-related effects depicted are derived from non-uremic models, and should not be interpreted as established mechanisms in CKD-MBD.

*The pathway (established).* RANKL binding to RANK recruits TRAF6 and activates Rac1-dependent NADPH oxidase, while mitochondrial ROS provide an additional oxidant source [15,16]. The resulting ROS burst is required for osteoclast differentiation and integrates with ERK, p38, JNK, AKT, and canonical NF-κB pathways, culminating in c-Fos and NFATc1 activation. NFATc1 then induces the osteoclast effector programme, including TRAP/Acp5, cathepsin K, MMP-9, carbonic anhydrase II, V-ATPase components, DC-STAMP, and the calcitonin receptor [15,16,78].

*NRF2 as a physiological brake (established).* RANKL/M-CSF signalling lowers the NRF2/KEAP1 ratio in osteoclast precursors, allowing the ROS rise required for differentiation. Experimental NRF2 activation reduces intracellular ROS and osteoclast formation, whereas NRF2 loss has the opposite effect [24,78]. Nrf2-deficient bone-marrow macrophages accumulate cytoplasmic and mitochondrial ROS and differentiate more readily, an effect reversible by antioxidant or NOX/mitochondrial ROS inhibition [25]. Pharmacological disruption of KEAP1-NRF2 interaction suppresses osteoclastogenesis and ovariectomy-induced bone loss [23], and a clinical-stage NRF2 activator has shown a similar anti-osteoclastic effect [79]. Collectively, these studies establish NRF2 as an important endogenous restraint on osteoclastogenesis.

*H_2_ actions on osteoclasts (established core finding).* In RAW264.7 cells and primary bone-marrow macrophages stimulated with RANKL, H_2_ inhibited osteoclast differentiation and resorption-pit formation [43]. It reduced intracellular ROS, NADPH oxidase and Rac1 activity, and suppressed MAPK, AKT, and NF-κB signalling, together with multiple osteoclast effector genes. These data place the effect upstream within the RANKL-Rac1/NOX-redox network, rather than at a single terminal oxidative product.

The anti-osteoclastic signal is supported by several in vivo models. HRW reduced osteoclast activation and bone loss in glucocorticoid-exposed zebrafish scales [45], and hydrogen water attenuated oxidative stress, osteoclastogenesis, and bone loss during hindlimb unloading [42]. In ageing periodontal tissue and high-fat-diet obesity, HRW reduced oxidative injury and alveolar osteoclast activity [46,47]. In ovariectomised rats, hydrogen water preserved femoral and vertebral bone-mineral measures, while reducing femoral oxidative stress [44]. Although heterogeneous, these models consistently support an antiresorptive effect.

*Relevance to CKD (hypothesised).* High-turnover ROD caused by SHPT is characterised by excessive RANKL-driven osteoclastogenesis in an inflammatory milieu. The pathways inhibited by H_2_ in non-uremic models—Rac1/NOX-derived ROS, MAPK, AKT, NF-κB, and, potentially, Ca^2+^/NFATc1, map directly onto this lesion. A testable prediction is that H_2_ will preferentially reduce resorption markers such as TRAP-5b and CTX in high-turnover CKD. In low-turnover disease, however, further osteoclast suppression could be neutral or harmful; this competing effect is examined in Section 5.6.

### 5.2. Osteoblasts and Osteoprogenitors: NRF2, Wnt/β-Catenin–FoxO and Mitochondrial Competence

**Figure 5:**
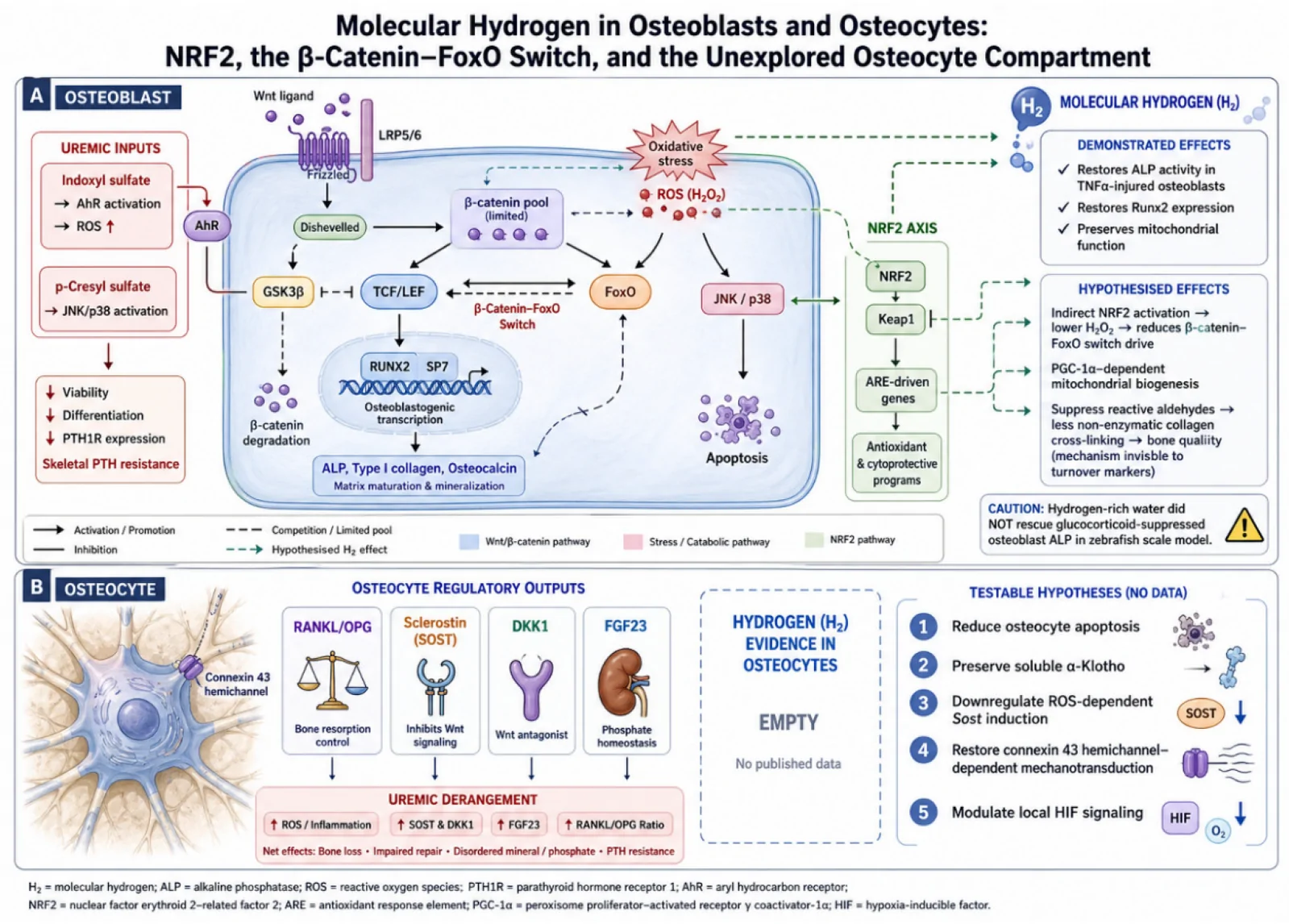
Molecular hydrogen in osteoblasts and osteocytes: NRF2, the β-catenin–FoxO switch, and the unexplored osteocyte compartment. Upper panel (osteoblast): Wnt ligand engagement of Frizzled/LRP5-6 stabilises β-catenin for TCF/LEF-dependent transcription of RUNX2 and SP7, driving alkaline phosphatase, type I collagen and osteocalcin expression. Oxidative stress activates FoxO, which competes with TCF for a limiting β-catenin pool, diverting Wnt signalling away from osteoblastogenesis; ROS additionally activate JNK/p38 to promote apoptosis, and GSK3β couples β-catenin and NRF2 regulation. Uremic inputs (indoxyl sulfate via AhR and ROS; p-cresyl sulfate via JNK/p38) suppress viability, differentiation and PTH1R expression, producing skeletal PTH resistance. Demonstrated H_2_ effects are marked: restoration of alkaline phosphatase activity and Runx2 expression in TNF-α–injured osteoblasts, with preserved mitochondrial function. Hypothesised effects are marked with dashed arrows: indirect NRF2 activation lowering the H_2_O_2_ pool that drives the β-catenin–FoxO switch; PGC-1α–dependent mitochondrial biogenesis; and suppression of reactive aldehyde generation with consequent reduction in non-enzymatic collagen cross-linking—a bone quality mechanism invisible to conventional turnover endpoints. A cautionary annotation records the contradictory finding that hydrogen-rich water did not rescue glucocorticoid-suppressed osteoblast alkaline phosphatase in the zebrafish scale model. Lower panel (osteocyte): the osteocyte regulatory outputs—RANKL/OPG, sclerostin, DKK1 and FGF23—and their uremic derangement, shown against an explicitly empty H_2_ evidence box. Five testable hypotheses are annotated: reduction of osteocyte apoptosis; preservation of soluble α-Klotho; downregulation of ROS-dependent Sost induction; restoration of connexin-43 hemichannel–dependent mechanotransduction; and modulation of local HIF signalling. No direct osteocyte data were identified in the literature searched; these pathways are presented as testable hypotheses and priorities for experimental validation.

The principal redox-sensitive mechanisms relevant to osteoblast function and their potential modulation by H_2_ are summarized in Figure 5.

*The pathway (established)*. Osteoblast differentiation requires canonical Wnt/β-catenin signalling, TCF/LEF-dependent induction of RUNX2 and SP7/Osterix, and subsequent expression of alkaline phosphatase, type I collagen, and osteocalcin. Oxidative stress disrupts this programme by activating FoxO, which competes with TCF for β-catenin [17,18], and by engaging stress kinases and apoptosis pathways. GSK3β provides additional redox-sensitive coupling between β-catenin and NRF2 signalling [17,85,86]. The ROS-FoxO–β-catenin axis also contributes to age- and oestrogen-deficiency-associated bone loss [19,20,21].

*The uremic overlay (established).* IS impairs osteoblast viability and differentiation, and can downregulate PTH1R, contributing to skeletal PTH resistance; pCS activates JNK and p38 MAPK in osteoblasts. These effects, together with metabolic acidosis and inflammatory signalling, create a low-anabolic environment in CKD. The resulting phenotype is compatible with the low-turnover pattern described in earlier CKD, although turnover cannot be inferred from PTH alone [90,91].

*H_2_ actions on osteoblasts (suggested).* In TNF-α-injured rat osteoblasts, H_2_ restored alkaline phosphatase activity and Runx2 expression while reducing oxidative and inflammatory injury and preserving mitochondrial function [48]. H_2_ also improved osteoblastic differentiation in a simulated-microgravity model [42]. In rabbit steroid-induced osteonecrosis, H_2_ reduced oxidative stress and apoptosis with histological protection [49], and HRS preserved bone volume and biomechanical properties in diabetic rats [50]. These studies support a potential osteoblast-protective effect, but do not establish a consistent anabolic action.

*Contradictory evidence.* In prednisolone-treated zebrafish scales, HRW prevented osteoclast activation, but did not rescue suppressed osteoblast alkaline phosphatase activity or bone repair [45]. This osteoclast-selective result is important because it limits any assumption that H_2_ is intrinsically anabolic. In low-turnover ROD, where remodelling activation is already reduced [90,91], the net effect will depend on whether osteoblast/osteocyte protection outweighs further suppression of osteoclast-mediated remodelling initiation.

*Mechanistic candidates for an osteoblast effect (hypothesised).* Three routes warrant direct testing: (i) modest NRF2 activation that increases HO-1, NQO1, and glutathione capacity and reduces diversion of β-catenin from TCF toward FoxO [13,32,85]; (ii) PGC-1α-associated improvement in mitochondrial biogenesis and bioenergetic capacity [87]; and (iii) suppression of lipid radical-chain reactions and reactive aldehyde formation [30,31], which could reduce collagen and matrix-protein carbonylation. The third mechanism is particularly relevant to CKD, because it could improve bone material quality without producing a large change in conventional turnover markers or DXA.

### 5.3. Osteocytes: Sclerostin, RANKL/OPG, FGF23–Klotho and Mechanotransduction (Figure 4)

*The pathway (established).* Osteocytes coordinate adult bone remodelling by supplying RANKL, producing sclerostin and DKK1, secreting FGF23, and transducing mechanical loading through cilia, integrin-based attachments, and connexin-43 hemichannels [21,51,52]. Osteocyte apoptosis can initiate targeted remodelling, and excessive oxidative stress promotes osteocyte death and impairs mechanotransduction [19,20,21].

*The uremic overlay (established).* Osteocytic Wnt/β-catenin repression occurs early in progressive ROD [83]. Sclerostin and FGF23 generally rise as kidney function declines, while Klotho decreases, altering mineral and redox signalling [69,70,71]. IS also increases osteocytic Sost and Dkk1 expression and changes the RANKL/OPG balance [65,67], linking retained uremic solutes to osteocyte-mediated remodelling signals.

H_2_ actions on osteocytes: a direct evidence gap. No study identified in this review examined H_2_ exposure in osteocytes or osteocyte-like lines such as MLO-Y4, IDG-SW3, or Ocy454, and no skeletal study measured sclerostin, DKK1, or osteocytic RANKL/OPG after H_2_ treatment. This gap is important because osteocytes integrate both formation and resorption signals, and could provide early biomarkers for a CKD trial.

*Testable osteocyte hypotheses.* First, H_2_ may reduce osteocyte apoptosis, consistent with its anti-apoptotic effects in steroid-induced osteonecrosis [49]. Second, H_2_ preserved renal Klotho expression after ischaemia-reperfusion injury [92], raising the possibility that soluble or skeletal α-Klotho could serve as a target-engagement biomarker in CKD. Third, reduction of ROS-dependent Sost induction could de-repress Wnt signalling. Fourth, redox-sensitive connexin-43 hemichannel function and mechanotransduction may be restored. Fifth, H_2_-related modulation of HIF signalling [13] could alter the response of osteocytes to their physiologically hypoxic environment. Each mechanism remains untested in bone, and should be labelled accordingly.

These proposals are mechanistic hypotheses, rather than demonstrated H_2_ effects. Existing osteocyte culture systems permit direct and relatively rapid testing under defined uremic conditions.

### 5.4. Marrow Stroma and the Osteoimmune Interface

Bone-marrow stromal cells balance osteogenic and adipogenic differentiation, and oxidative stress together with inflammatory cytokines can shift this balance toward adipogenesis. In CKD, the same inflammatory milieu that impairs osteoblast supply also promotes RANKL-dependent resorption through IL-1β, IL-6, TNF-α, and MCP-1 [58,72].

H_2_ has anti-inflammatory effects in kidney-injury models relevant to this interface. HRW reduced renal NF-κB activation and pro-inflammatory cytokines in spontaneously hypertensive rats [93], while H_2_ treatment in septic renal injury reduced IL-6 and TNF-α, increased IL-10 and TGF-β, and favoured M2 macrophage polarisation [94]. Extrapolating these effects to uremic bone is biologically plausible, but none of these studies measured skeletal outcomes.

### 5.5. The Hormetic Problem: Why “More Antioxidant” Is Not Better in Bone

Redox-directed therapy in bone must account for a non-monotonic NRF2 dose–response. NRF2 deficiency increases osteoclastogenesis and bone resorption [24,25,78], whereas excessive NRF2 activation can also impair skeletal homeostasis: Keap1 disruption produces osteopenia and defective osteoblast differentiation, while more moderate NRF2 activation in Keap1 heterozygosity can increase mineral apposition and bone formation [79,95,96]. Other NRF2-activated models similarly show suppression of both osteoclastogenesis and osteoblastogenesis, with net bone hypoplasia when osteoblast inhibition predominates [97].

These observations support the existence of a redox-response window, rather than a simple ‘more antioxidant is better’ relationship. The mixed clinical experience with potent NRF2 activators in CKD is consistent with the broader difficulty of pharmacologically manipulating this pathway, but adverse or neutral renal outcomes cannot be attributed specifically to a skeletal hormetic mechanism [98,99].

H_2_ is therefore of interest as a comparatively mild, indirect redox modulator, rather than as a forced NRF2 activator. It may reduce upstream oxidative burden while preserving part of physiological redox signalling [13,27,30]. This signal-sparing concept is testable: if clinically achievable H_2_ exposure does not measurably alter skeletal redox or target-engagement biomarkers, the same modest pharmacology that motivates the approach would also explain lack of efficacy.

### 5.6. Turnover-State-Dependent Actions of H_2_: A Bidirectional Remodelling-Normaliser Hypothesis for Low- Versus High-Turnover Renal Osteodystrophy

The lineage-specific evidence suggests a testable turnover-state model. ROD spans low-turnover adynamic bone disease (ABD), normal turnover, and high-turnover osteitis fibrosa. Excess ROS can suppress osteoblast differentiation [13,17] while simultaneously serving as an obligatory signal for RANKL-driven osteoclastogenesis [15,16]. H_2_ might therefore reduce pathological deviation from normal turnover, rather than shift remodelling uniformly in one direction. This ‘bidirectional remodelling–normaliser’ is a hypothesis, not an established effect, and its key prediction is a prespecified H_2_ × turnover-state interaction.

*Low-turnover disease: potential release of uremic suppression (hypothesised).* ABD reflects more than low PTH. Uremic toxins, inflammation, oxidative stress, and mitochondrial injury can directly suppress osteoblast-lineage function. IS induces oxidative stress and PTH resistance in osteoblasts, attenuating the PTH1R-Gsα-cAMP-PKA-CREB pathway [61,63], while mitochondrial dysfunction and mitophagy blockade have been demonstrated in CKD-MBD bone [100]. In osteocytes, IS lowers RANKL/OPG and increases sclerostin and DKK1, changes that are reversed by PTH [101]. H_2_ could, theoretically, improve mitochondrial competence, PTH responsiveness, and osteocyte Wnt-antagonist output. A discriminating experiment would hold PTH constant and test whether H_2_ increases cAMP/CREB and RUNX2/Osterix responses in uremic osteoblasts [48]. These predicted rescue mechanisms remain unproven.

*The adynamic-bone paradox.* The most reproducible skeletal action of H_2_ is inhibition of osteoclastogenesis through reduction of ROS and suppression of MAPK, AKT, and NF-κB signalling [42,43,45]. In established ABD, further osteoclast suppression could reduce remodelling activation and thereby limit coupled osteoblast recruitment. Consequently, benefit in low-turnover disease cannot be assumed; it requires the osteoblast/osteocyte rescue effect to exceed the anti-osteoclastic effect. This competing biology is why turnover-state stratification and coupled formation/resorption endpoints are essential.

*High-turnover disease: strongest mechanistic alignment (hypothesised).* In SHPT, persistent PTH1R signalling in osteoblast-lineage cells and osteocytes raises RANKL and lowers OPG, activating the TRAF6-Rac1-NOX-ROS-MAPK/NF-κB-NFATc1 cascade. H_2_ could act upstream by reducing oxidative/inflammatory signals that influence RANKL/OPG [101] and downstream by attenuating the RANKL-induced ROS burst in osteoclast precursors [43]. RANKL inhibition improves cortical and resorptive abnormalities in adenine-induced CKD [96], supporting the pathological relevance of this axis. A uremic H_2_ experiment should therefore determine whether osteoclast NFATc1, cathepsin K, and cortical porosity fall despite unchanged PTH, which would indicate a skeletal, rather than parathyroid-mediated, effect.

*Why opposite effects are biologically plausible (hypothesised).* The relevant variable may be lineage-specific pathological redox load, rather than turnover itself. Excess ROS inhibits osteoblast differentiation but supports osteoclastogenesis. A modulator that preferentially limits highly damaging oxidant chemistry while sparing physiological signalling [27,30,31] could therefore have different net effects across lineages and turnover states. The defining prediction is not guaranteed stimulation of low-turnover bone, but a turnover-dependent response with minimal disturbance of normal remodelling.

**Figure 6:**
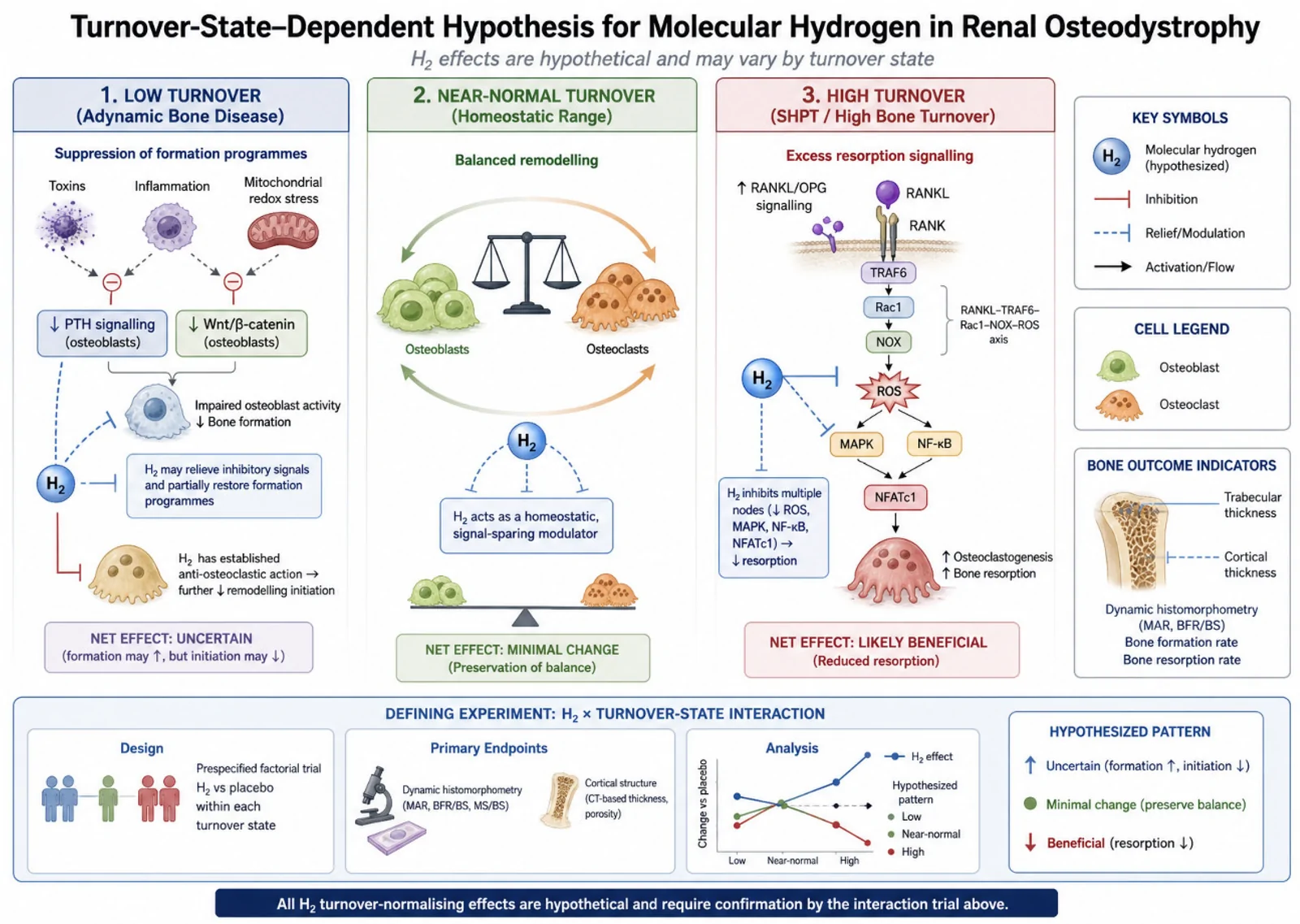
Turnover-state-dependent hypothesis for molecular hydrogen in renal osteodystrophy. Low-turnover/adynamic bone disease is shown with toxin-, inflammation-, and mitochondrial-redox-mediated suppression of PTH/Wnt osteoblast programmes. H_2_ is hypothesised to relieve some of these inhibitory signals, but its established anti-osteoclastic action could further reduce remodelling initiation; the net effect is therefore uncertain. In high-turnover SHPT, increased RANKL/OPG signalling engages the RANKL-TRAF6-Rac1-NOX-ROS-MAPK/NF-κB-NFATc1 cascade, providing a stronger mechanistic rationale for H_2_-mediated suppression of resorption. Near-normal turnover is predicted to show limited change if H_2_ acts as a homeostatic, signal-sparing modulator. All turnover-normalising effects remain hypothetical; the defining experiment is a prespecified H_2_ × turnover-state interaction using dynamic histomorphometry and cortical-structure endpoints.

A longitudinal implication is that the dominant skeletal lesion may change during CKD progression [61,102]. H_2_ could, theoretically, preserve osteoblast responsiveness earlier while restraining RANKL/ROS-dependent resorption after SHPT becomes established. This should be tested in normal-, low-, and high-turnover uremic models randomised to H_2_ or vehicle, using dynamic histomorphometry and cortical porosity with a prespecified H_2_ × turnover-state interaction. Source-matched ex vivo cells could help distinguish intrinsic skeletal responses from systemic confounding. Until such studies are performed, the bidirectional-normaliser model remains a testable framework, rather than a description of demonstrated H_2_ behaviour (Figure 6).

## 6. Preclinical Evidence for H_2_ in Skeletal Disease

Table 2 summarises the preclinical skeletal evidence. Four features are most relevant to interpretation.

*Antiresorptive signal.* Across RANKL-stimulated cells, ovariectomy, hindlimb unloading, glucocorticoid exposure, periodontal ageing, obesity, and diabetic bone disease, H_2_ has repeatedly been associated with reduced osteoclast activity or preservation of bone mass [42,43,44,45,46,47,48,49,50]. The consistency across diverse injury models supports biological plausibility, although study quality, species, exposure methods, and publication bias limit certainty.

*Osteoblast signal.* Osteoblast rescue has been reported in TNF-α-injured cells and simulated microgravity [42,48], but was not observed in glucocorticoid-exposed zebrafish scales [45]. An anabolic effect should therefore be considered model-dependent and unresolved.

*Uremic evidence gap.* H_2_ has been studied in experimental kidney disease for renal and cardiovascular outcomes, but no intervention study identified here assessed skeletal endpoints in a CKD/uremic model. Because uremic bone differs from conventional osteoporosis models in turnover, mineralisation, endocrine signalling, and matrix chemistry, direct extrapolation is limited.

*Methodological limitations.* H_2_ concentration and delivered dose are inconsistently reported, and losses related to preparation, storage, and administration are rarely quantified. Randomisation, allocation concealment, blinded outcome assessment, and sample-size justification are variably described. Several models are also short, relative to the time scale of bone remodelling. These limitations increase the risk of effect-size inflation and publication bias.

## 7. Clinical Evidence for H_2_ in CKD and Dialysis

Table 3 summarises the human evidence. Clinical experience is concentrated in dialysis populations, is sparse in non-dialysis CKD, and has not included bone-specific outcomes.

*H_2_-based dialysis studies.* Early haemodialysis studies using electrolysis-derived dissolved H_2_ established feasibility and reported changes in oxidative-stress-related measures [34,36]. A separate peritoneal dialysis study used albumin redox state to demonstrate a local/systemic oxidative-stress signal after dissolved H_2_ exposure [35]. In haemodialysis, a 12-month interim analysis of 262 patients reported less severe fatigue and pruritus and lower antihypertensive medication use without a difference in dialysis adequacy [37]. The subsequent prospective observational cohort of 309 patients reported a favourable composite clinical outcome with E-HD [38]. A later review summarised the clinical H_2_ experience and highlighted unresolved mechanistic and implementation questions [103]. Independent electrolysed-reduced-water studies have also reported effects on dialysis-associated oxidative stress, erythrocyte injury, and T-cell indices [104,105,106].

*Critical appraisal.* The clinical signal is preliminary but not definitive. The pivotal E-HD outcome study was prospective but non-randomised and non-blinded, with treatment determined by centre capability, rather than individual random allocation. This design permits centre effects and residual confounding, while composite outcomes and practice-specific dialysis protocols limit generalisability. The available evidence therefore supports feasibility and biological activity, but does not establish efficacy for CKD-MBD or skeletal outcomes.

*Oral formulations.* The first-in-human HRCC study enrolled 16 participants with metabolic syndrome at three dose levels for four weeks; no major safety signal was reported, triglycerides decreased, and quality-of-life measures were unchanged [39]. The uncontrolled design, small sample, short duration, and non-CKD population mean that the study establishes preliminary tolerability only.

Bone-specific clinical evidence. No CKD H_2_ study identified in this review reported bone-specific alkaline phosphatase, TRAP-5b, CTX, P1NP, sclerostin, FGF23, BMD, histomorphometry, or fracture as an endpoint. The registry search, likewise, did not identify a completed CKD H_2_ trial with a skeletal endpoint [13]. Any proposed renal bone benefit must therefore be regarded as a hypothesis derived from non-skeletal models and adjacent redox biology.

## 8. The Bone–Vascular Axis and the Compartment-Specificity Problem

Vascular calcification and skeletal fragility coexist in CKD. Vascular smooth muscle cells (VSMCs) can undergo active osteogenic transdifferentiation in response to hyperphosphataemia, calciprotein particles, inflammation, oxidative stress, and Klotho deficiency [68,69,70,71,107,108]. IS-induced ROS is one mechanism capable of promoting this osteogenic vascular switch [62,108].

Systemic redox modulation could therefore influence both bone and vasculature. Supporting evidence is indirect: molecular hydrogen stabilised atherosclerotic plaque in LDL-receptor-knockout mice [109], and HRW reduced renal NF-κB activation in hypertensive rats [93]. Neither model establishes vascular benefit in CKD-MBD.

**Figure 7:**
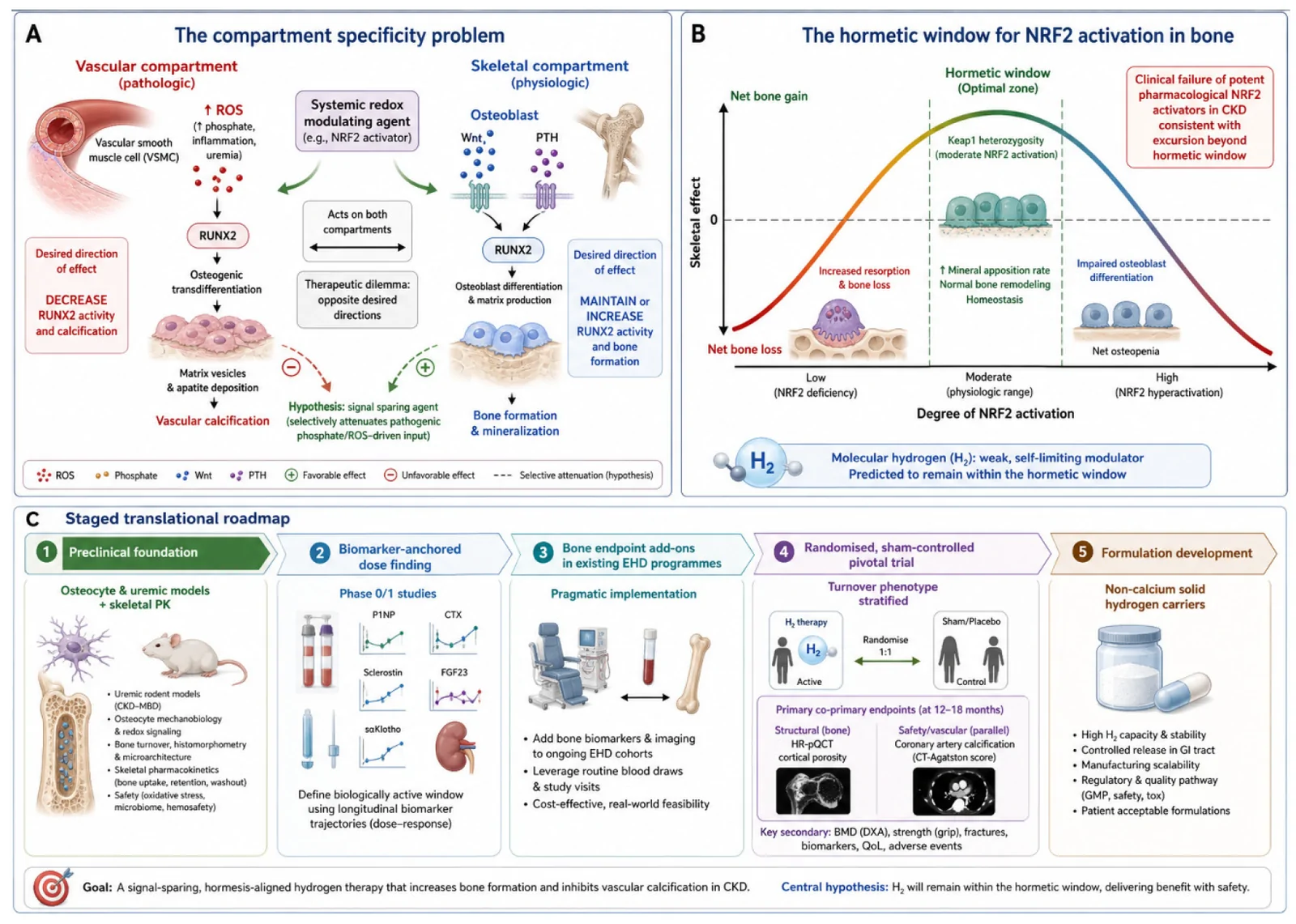
Bone–vascular compartment specificity, the redox-response window, and a translational roadmap. Panel (**A**) contrasts pathological ROS/phosphate-driven RUNX2 activation in vascular smooth muscle cells with physiological Wnt/PTH-dependent RUNX2 activity in osteoblasts, illustrating why systemic redox modulation requires parallel skeletal and vascular assessment. Panel (**B**) depicts the non-monotonic relationship between NRF2 activity and bone remodelling: insufficient NRF2 favours oxidative osteoclastogenesis, whereas excessive activation can inhibit osteoblast differentiation; H_2_ is positioned as a hypothesised mild, signal-sparing modulator, rather than a forced NRF2 activator. Panel (**C**) proposes sequential development: (1) osteocyte and uremic-bone studies with skeletal pharmacokinetics; (2) biomarker-anchored dose/exposure studies; (3) skeletal add-ons to dialysis cohorts; (4) a turnover-stratified randomised trial with one prespecified primary mechanistic endpoint and secondary HR-pQCT plus vascular-calcification outcomes; and (5) development of non-calcium solid H_2_ carriers.

This creates a compartment-specificity problem. RUNX2 induction is undesirable when it reflects pathological osteogenic conversion of VSMCs, but RUNX2 is essential for normal osteoblast differentiation. A systemic redox intervention will reach both compartments. Signal-sparing modulation may preferentially attenuate pathological phosphate/ROS-driven vascular signalling while preserving physiological Wnt- and PTH-dependent osteoblast programmes, but this remains a hypothesis. Clinical studies should therefore assess skeletal and vascular outcomes in parallel, particularly when the H_2_ formulation also contains a calcium carrier (Figure 7).

Beyond a ROS-centred model, extracellular-matrix (ECM) turnover provides an additional redox-sensitive axis relevant to CKD-MBD. Matrix metalloproteinases (MMPs), particularly MMP-2 and MMP-9, regulate ECM degradation and remodelling and participate in the transition from oxidative injury and inflammation to renal fibrosis; MMP-9 is also part of the osteoclast effector programme discussed in Section 5.1, providing a conceptual link between renal and skeletal matrix remodelling under oxidative stress [110]. This reference is cited only as a biologically relevant framework: it does not provide direct evidence for a beneficial effect of H_2_ on renal fibrosis or MMP activity, and H_2_ action on ECM/MMP biology in renal bone disease remains a mechanistic hypothesis (Figure 3).

## 9. Clinical Implications

Five practical implications follow from the current evidence (Table 4).

*H*_2_ *should be studied as an adjunct, not a replacement.* The available evidence does not support substituting H_2_ for phosphate management, vitamin D therapy, PTH control, or established osteoporosis treatment when indicated [8,9,10]. Any future role would be an additive to guideline-directed CKD-MBD care.

*Maintenance haemodialysis is the most practical initial clinical setting.* This population has high fracture and oxidative-stress burdens, routine biochemical monitoring, and an existing extracorporeal circuit through which H_2_ can be delivered without additional fluid intake [4,34,37,38]. The reported effect of high-H_2_ dialysate on albumin-bound indoxyl sulfate provides an additional, bone-relevant mechanistic rationale that requires clinical confirmation [73].

*Turnover phenotype should be prespecified.* Direct preclinical evidence is strongest for inhibition of osteoclastogenesis, which aligns most clearly with high-turnover SHPT. In low-turnover/adynamic bone, the net effect is uncertain because potential osteoblast or osteocyte rescue competes with further suppression of osteoclast-mediated remodelling. Because ABD is common across CKD and dialysis populations [90,91], PTH, bone-specific ALP, and TRAP-5b should be used to enrich probable turnover strata, rather than to claim definitive histological classification. Bone histomorphometry remains the reference method and should be incorporated in a mechanistic substudy where feasible.

*Calcium exposure from HRCC must be treated as an intervention variable.* In non-dialysis CKD, solid H_2_ delivery is attractive because it avoids fluid loading [13,39,40], but a calcium-carbonate carrier directly affects the calcium-balance and vascular-calcification issues central to CKD-MBD [8,9]. Trials should report elemental calcium per dose, total concurrent calcium exposure, calcium and PTH responses, and vascular calcification. A non-calcium H_2_ carrier would be preferable for advanced CKD, and should be prioritised for formulation development.

*Early trials should use mechanistic and structural endpoints, rather than fracture.* Feasible outcomes include systemic oxidative stress markers, bone turnover markers, sclerostin, FGF23, soluble α-Klotho, and HR-pQCT measures of cortical porosity and trabecular microarchitecture [52,53]. Fracture prevention should remain a later-stage efficacy endpoint once target engagement and skeletal safety have been established. In advanced CKD, bone-specific alkaline phosphatase (BALP) and TRAP-5b are the preferred turnover markers because CTX and total P1NP are influenced by reduced renal clearance; FGF23, sclerostin, and soluble α-Klotho should be regarded as exploratory CKD-MBD/systemic biomarkers, rather than validated skeletal H_2_ target-engagement markers.

## 10. Knowledge Gaps and Future Directions

Eight research priorities are proposed, ordered broadly from mechanistic tractability to clinical translation.

(i) Define osteocyte responses to H_2_. Expose MLO-Y4, IDG-SW3, and Ocy454 cells to H_2_ under uremic conditions (IS, pCS, and high phosphate) and measure SOST, DKK1, RANKL/OPG, FGF23, apoptosis, connexin-43 hemichannel activity, and mechanoresponsiveness. This directly addresses the largest lineage-specific evidence gap.
(ii) Test H_2_ in uremic bone models. Compare well-characterised H_2_ delivery methods in 5/6 nephrectomy, adenine-induced CKD, and the Cy/+ rat using dynamic histomorphometry, µCT, biomechanical testing, and matrix-quality measures. These studies should include turnover-state phenotyping, rather than a single CKD-versus-control contrast.
(iii) Establish skeletal pharmacokinetics. Measure H_2_ exposure in blood, marrow, mineralised bone, and, where technically possible, the lacuno–canalicular compartment after each delivery modality. Tissue access is plausible, but currently unverified.
(iv) Test the NFAT hypothesis. Determine whether H_2_ suppresses RANKL-induced Ca^2+^ oscillations, calcineurin activity, and NFATc1 autoamplification in osteoclast precursors, and whether oxidised phospholipid mediators are involved [30,31]. This experiment could connect H_2_ chemistry directly to the established anti-osteoclastic phenotype.
(v) Define the exposure-response window. Test a broad range of H_2_ exposures in osteoblast and osteoclast systems, including markers of reductive stress, to determine whether responses are biphasic and how they relate to NRF2 activity [95,96].
(vi) Add skeletal endpoints to existing dialysis programmes. Where E-HD is already in use, prospective collection of bone turnover markers, sclerostin, FGF23, soluble α-Klotho, and HR-pQCT could provide an efficient first assessment of human skeletal target engagement without claiming causal efficacy.
(vii) Conduct a turnover-stratified randomised trial. An early-phase double-blind, sham-controlled trial of H_2_-enriched versus conventional dialysate should prespecify one primary mechanistic endpoint, such as a validated bone-turnover or systemic-redox measure, and stratify by probable turnover phenotype. HR-pQCT cortical porosity, other bone markers, and vascular calcification should be secondary or exploratory endpoints. The protocol should verify H_2_ exposure and target engagement, standardise sampling relative to dialysis, control major CKD-MBD therapies and comorbidities, and include prespecified safety monitoring. A tetracycline-labelled bone-biopsy substudy would provide the most definitive turnover classification where feasible.
(viii) Standardise formulation and exposure reporting. Non-calcium solid H_2_ carriers should be developed for CKD, and studies should report dissolved H_2_ concentration, delivered mass, route, exposure duration and frequency, preparation and storage conditions, time to administration, measurement method, and blood or tissue concentrations, where available. HRW, HRS, E-HD, and solid H_2_ formulations should be treated as pharmacologically distinct exposures, rather than interchangeable categories.

## 11. Limitations

The principal limitation of this review is the indirectness of the evidence base.

The review is narrative, rather than formally systematic. It was not registered, did not use duplicate independent screening, and did not apply a formal risk-of-bias instrument; selection and interpretation bias, therefore, remain possible, despite the structured search and explicit evidence grading.

Direct evidence at the intersection of H_2_, CKD, and skeletal outcomes was not identified. The proposed mechanisms therefore depend on triangulation across uremic bone biology, non-uremic skeletal H_2_ models, and H_2_ studies in kidney disease, each with potential for translational failure.

Publication and commercial bias may also affect the H_2_ literature. Clinical experience is concentrated within a limited number of programmes, and negative preclinical findings may be under-represented. These considerations reinforce the need for independent replication and transparent disclosure.

H_2_ dose and exposure are poorly standardised across studies, limiting quantitative comparison and preventing a clinically defensible dose recommendation.

Finally, commonly used skeletal models, including zebrafish scales, hindlimb unloading, ovariectomy, diabetes, and rabbit osteonecrosis, do not reproduce the endocrine, mineral, and matrix environment of human uremic bone. Their findings should therefore be treated as mechanistic support, rather than direct evidence for CKD-MBD.

## 12. Conclusions

Oxidative and redox-signalling disturbances are biologically plausible contributors to renal bone disease because uremic toxins, inflammation, mineral dysregulation, and dialysis converge on pathways that control osteoclasts, osteoblast-lineage cells, and osteocytes. Molecular hydrogen is of interest because its small size and proposed signal-sparing redox actions may differ from broad antioxidant or potent NRF2-activating strategies.

The evidence remains indirect. H_2_ reproducibly suppresses osteoclastogenesis in non-uremic models, whereas osteoblast responses are inconsistent and direct osteocyte data are absent. No CKD H_2_ study identified here included a bone-specific endpoint. The strongest mechanistic case therefore concerns high-turnover disease, where H_2_-mediated suppression of the RANKL-ROS-NFATc1 axis aligns with the pathological lesion. In low-turnover/adynamic bone, benefit is uncertain because putative osteoblast/osteocyte rescue could be offset by further inhibition of remodelling initiation. Calcium-containing solid formulations introduce an additional CKD-specific vascular and calcium-balance concern.

H_2_ should therefore be regarded as a testable adjunctive strategy, rather than an established CKD-MBD treatment. Priority studies should establish skeletal pharmacokinetics, directly test H_2_ in uremic bone, prespecify turnover-state interactions, standardise exposure, and assess skeletal and vascular outcomes in parallel. Independent, adequately controlled clinical trials are required before therapeutic claims can be made, with particular caution in low-turnover disease.

## Figures and Tables

**Table 1:** Comparison of molecular hydrogen delivery modalities for CKD and renal bone disease.

Modality	Typical Delivery	Pharmacokinetic Profile	Advantages in CKD	Limitations and CKD-Specific Cautions	Suitability for a Bone-Endpoint Trial
H_2_-enriched dialysate (E-HD) [34,36,37,38]	Dissolved H_2_ (≈30–80 ppb in studied systems) generated by water electrolysis, delivered across the dialyser for the full session	Sustained systemic exposure over 4–5 h, 3×/week; no fluid load	Exploits existing circuit; zero patient burden; no added fluid; longest clinical dataset; may enhance dissociation of albumin-bound indoxyl sulfate	Restricted to HD; requires dedicated equipment; interdialytic exposure gap; current outcome evidence is predominantly observational	High feasibility for a bone-endpoint trial; sham-controlled comparison is technically plausible
Inhaled H_2_ gas/H_2_–O_2_ mixture [27,88]	1–4% H_2_ in air, or H_2_–O_2_ blends, via mask or nasal cannula	Rapid arterial rise; exposure limited to inhalation period	Fast onset; precise dose control; useful for acute mechanistic studies	Equipment and supervision required; poor adherence for chronic use; institutional flammability concerns, despite sub-LEL concentrations; impractical for ambulatory CKD	Low for chronic bone endpoints; suitable for short mechanistic/PK substudies
Oral hydrogen-rich water (HRW) [44,46,47]	Saturated or near-saturated water (up to ≈1.6 ppm), typically several hundred mL to >1 L daily	Sharp, short post-ingestion peak; rapid decay; highly container- and handling-dependent	Inexpensive; widely available; largest general human dataset	Fluid volume conflicts directly with interdialytic weight-gain limits and CKD fluid restriction; pulsatile dosing; substantial inter-product variability in delivered H_2_	Low in dialysis; possible in CKD G3 with careful fluid accounting
Hydrogen-rich saline (HRS) [49,50]	Saturated saline, intraperitoneal or intravenous	Controlled systemic dosing	Precise preclinical dosing; used in several preclinical bone studies (delivery modalities vary across studies; see Table 2)	Parenteral route; sodium load; not a practical chronic clinical modality	Preclinical only; essential for uremic bone-model work
Oral solid hydrogen capsules (OSHC), incl. hydrogen-rich coral calcium (HRCC) [39,40]	Solid formulation generating H_2_ in situ in the gastrointestinal tract	Slower, more sustained release than HRW; no fluid load	Fluid-sparing ambulatory option for non-dialysis CKD; storage-stable; preliminary human tolerability data available	Calcium-carbonate carriers add elemental calcium and may affect calcium balance, PTH, and vascular calcification. Elemental calcium exposure should be quantified and monitored; non-calcium carriers are preferable for future CKD studies	Potentially suitable for non-dialysis CKD if calcium exposure and vascular safety are prospectively addressed

**Table 2:** Preclinical evidence for molecular hydrogen in skeletal and bone-cell models.

Model/System	H_2_ Modality and Exposure	Principal Skeletal Findings	Signalling Mechanism Reported	Evidence Tier	Ref.
RAW264.7 cells and primary bone-marrow macrophages, RANKL-stimulated	H_2_ incubation (dissolved H_2_ medium)	Prevented osteoclast differentiation and resorption-pit formation; reduced osteoclast effector-gene expression	↓ Intracellular ROS; ↓ NADPH oxidase activity; ↓ Rac1 activity; inactivation of MAPK, AKT and NF-κB	Established (core anti-osteoclastic effect); Suggested (Rac1 specificity)	[43]
Cultured rat osteoblasts, TNF-α–injured	H_2_-rich medium	Reversed suppression of alkaline phosphatase activity and *Runx2* mRNA; alleviated cell injury	↓ Oxidative stress and inflammatory signalling; preservation of mitochondrial function	Suggested	[48]
Hindlimb-suspension rats and rotary-wall-vessel MC3T3-E1/RAW264.7 (modelled microgravity)	Hydrogen water (in vivo); hydrogen-rich medium (in vitro)	Alleviated bone loss; restored osteoblastic differentiation; suppressed osteoclast differentiation and osteoclastogenesis	Abatement of oxidative stress	Suggested	[42]
Ovariectomised rats (post-menopausal osteoporosis model)	Hydrogen water, oral	Prevented osteopenia; preserved bone mineral content and density at femur and vertebrae	Reduced femoral oxidative stress	Suggested	[44]
Prednisolone-treated zebrafish scales (glucocorticoid-induced osteoporosis)	Hydrogen-rich water immersion	Prevented osteoclast activation and bone loss, but did not rescue glucocorticoid-suppressed osteoblast ALP activity or bone repair	Osteoclast-selective antioxidant effect	Established (osteoclast-selective pattern)	[45]
Streptozotocin-induced diabetic rats	Hydrogen-rich saline, systemic	Preserved bone volume; improved bone structural and material properties; reduced fracture risk indices	Antioxidant; safe and well tolerated	Suggested	[50]
Steroid-induced osteonecrosis, rabbit femoral head	Hydrogen-rich saline	Histological protection against osteonecrosis	↓ Oxidative stress; ↓ apoptosis	Suggested	[49]
Ageing periodontal tissue, rats	Hydrogen-rich water, drinking	Reduced alveolar bone loss (cemento-enamel junction to alveolar crest distance); fewer TRAP-positive osteoclasts	Reduced age-related oxidative damage	Suggested	[46]
High-fat-diet obese rats (Fischer 344)	Hydrogen-rich water, drinking	Reduced alveolar bone resorption; higher alveolar bone density on µCT; suppressed weight gain	Gingival 8-hydroxydeoxyguanosine	Suggested	[47]
Uremic bone models (5/6 nephrectomy, adenine CKD, Cy/+ rat)	—	No H_2_ skeletal study in a CKD/uremic model identified in the review search	—	Evidence gap	—
Osteocyte systems (MLO-Y4, IDG-SW3, Ocy454)	—	No H_2_ osteocyte study identified in the review search	—	Evidence gap	—

Abbreviations as in the abbreviation list. “Evidence tier” reflects the grading scheme defined in Section 2 and Table 2, ↓ means: decrease.

**Table 3:** Clinical studies of molecular hydrogen in CKD and dialysis populations, with assessment of bone relevance.

Study Design and Population	H_2_ Modality	Reported Outcomes	Bone Endpoints	Critical Appraisal	Ref.
Clinical trial, maintenance HD patients	Dissolved dihydrogen produced by water electrolysis in dialysate	Feasibility established; reductions in oxidative-stress markers; favourable hemodynamic signals	None	Small, early-phase, single-system; no comparator randomization	[34]
Clinical study, HD patients	Electrolysed water in HD	Biological effects on oxidative-stress and inflammatory indices	None	Exploratory; limited sample	[36]
Mechanistic clinical study, HD patients	Electrolysed H_2_-containing dialysis water	Changes in oxidative-stress-related and inflammatory/physiological indices	None	Supportive mechanistic evidence; small and exploratory	[36]
Prospective observational interim analysis; 262 HD patients, 12 months	H_2_-enriched dialysate	Less severe fatigue and pruritus; lower antihypertensive medication use; no difference in dialysis adequacy	None	Non-randomised and non-blinded; allocation by centre; residual confounding possible	[37]
Prospective observational study; 309 patients (161 E-HD, 148 conventional HD)	H_2_-enriched dialysate	Improved composite clinical outcome in E-HD group	None	Pivotal but observational, non-randomised and non-blinded; Japanese practice setting; residual confounding possible	[38]
Clinical/review evidence, chronic dialysis	H_2_/electrolysed-reduced-water dialysis approaches	Reported oxidative-stress, symptom, cellular, and physiological signals across heterogeneous studies	None	Heterogeneous designs and exposures; no skeletal endpoints	[103,104,105,106]
Interventional studies, ESRD on chronic HD	Electrolysed-reduced water dialysate	Reduced HD-induced oxidative stress; reduced erythrocyte impairment; improved T-cell damage indices	None	Independent group; mechanistically supportive	[104,105,106]
Experimental/translational peritoneal dialysis	Dissolved H_2_ in PD solution	Preserved mesothelial cells and peritoneal membrane integrity	None	Preclinical/translational; no skeletal endpoint	[89]
In vitro dialysate chemistry	High dissolved-H_2_ dialysate	Facilitated dissociation of indoxyl sulfate from albumin	Indirect bone relevance: IS is directly osteotoxic	Single study; requires replication and in vivo confirmation of enhanced clearance	[73]
Prospective dose-ranging case series; 16 patients with metabolic syndrome (Taiwan), 4 weeks; NCT05196295	Oral hydrogen-rich coral calcium (HRCC) capsules, low/medium/high dose	Primary safety endpoint met, no obvious adverse effects; significant triglyceride reduction; no change in quality of life	None	Small, uncontrolled, short duration; establishes tolerability only; calcium carrier load not analysed in a CKD context	[39]
Any H_2_ study in CKD with a skeletal endpoint identified in the review search	—	—	None	Direct evidence gap	—

**Table 4:** Critical evidence summary: current certainty and the study required to advance each question at the CKD–bone–H_2_ intersection.

Question	Current Evidence	Certainty	Required Next Study	References
Does H_2_ modify oxidative stress in dialysis?	Small, heterogeneous clinical and translational studies	Low	Independent randomised trial	[34,38,89]
Does H_2_ suppress osteoclastogenesis?	Multiple non-uremic preclinical models	Moderate (preclinical)	Uremic bone models	[42,43,45,50]
Does H_2_ improve osteoblast function?	Conflicting, model-specific findings	Very low	Dose–response studies under uremic conditions	[45,48,49]
Does H_2_ affect osteocytes?	No direct evidence identified	Absent	Osteocyte culture and uremic models	None
Does H_2_ prevent fracture in CKD?	No evidence	Absent	Later-stage outcome trials	None

## Data Availability

No new data were created or analysed in this study. Data sharing is not applicable to this article.

## Supplementary Materials

The following supporting information can be downloaded at: https://www.mdpi.com/article/10.3390/antiox15091137/s1. Supplementary Table S1. Scope of the review defined by a PICOTS framework with inclusion and exclusion criteria; Supplementary Table S2. Database specific Boolean search strategies.

## Abbreviations

ALP, alkaline phosphatase; ARE, antioxidant response element; BMD, bone mineral density; BSAP, bone-specific alkaline phosphatase; CKD, chronic kidney disease; CKD-MBD, chronic kidney disease–mineral and bone disorder; CTX, C-terminal telopeptide of type I collagen; DKK1, Dickkopf-1; DXA, dual-energy X-ray absorptiometry; E-HD, electrolysed-water haemodialysis (H_2_-enriched dialysate); FGF23, fibroblast growth factor 23; FoxO, forkhead box O; GPx, glutathione peroxidase; GSK3β, glycogen synthase kinase-3β; H_2_, molecular hydrogen; HD, haemodialysis; HIF, hypoxia-inducible factor; HO-1, haem oxygenase-1; HR-pQCT, high-resolution peripheral quantitative computed tomography; HRCC, hydrogen-rich coral calcium; HRS, hydrogen-rich saline; HRW, hydrogen-renalrich water; IS, indoxyl sulfate; KEAP1, Kelch-like ECH-associated protein 1; MAPK, mitogen-activated protein kinase; MDA, malondialdehyde; MPO, myeloperoxidase; NFATc1, nuclear factor of activated T cell cytoplasmic 1; NF-κB, nuclear factor kappa-light-chain-enhancer of activated B cells; NOX, NADPH oxidase; NQO1, NAD(P)H quinone dehydrogenase 1; NRF2, nuclear factor erythroid 2-related factor 2; 8-OHdG, 8-hydroxy-2′-deoxyguanosine; OPG, osteoprotegerin; OSHC, oral solid hydrogen capsule; P1NP, procollagen type I N-terminal propeptide; pCS, *p*-cresyl sulfate; PD, peritoneal dialysis; PGC-1α, peroxisome proliferator-activated receptor gamma coactivator 1-α; PHD, prolyl hydroxylase domain enzyme; PTH, parathyroid hormone; RANK(L), receptor activator of nuclear factor-κB (ligand); RNS, reactive nitrogen species; ROD, renal osteodystrophy; ROS, reactive oxygen species; RUNX2, runt-related transcription factor 2; SHPT, secondary hyperparathyroidism; SOD, superoxide dismutase; SOST, sclerostin gene; TCF, T-cell factor; TNF-α, tumour necrosis factor-α; TRAF6, TNF receptor-associated factor 6; TRAP-5b, tartrate-resistant acid phosphatase 5b; VSMC, vascular smooth muscle cell.
