# Supplementary material for: Molecular Hydrogen and the Uremic Skeleton: A Critical Review and Turnover-State-Dependent Redox Hypothesis in CKD-MBD
Source: Antioxidants (Basel). 2026 Sep 8;15(9):1137. doi: 10.3390/antiox15091137 (PMC13603868; doi:10.3390/antiox15091137)
Supplement: Supplementary file 1 [file antioxidants-15-01137-s001.zip › antioxidants-4536485-supplementary.pdf]

Supplementary data

Methods: Scope, Search Strategy and Evidence Appraisal

The review scope, expressed in a PICOTS framework with explicit inclusion and exclusion criteria, is presented in supplementary table 1.
Database-specific Boolean search strategies for PubMed/MEDLINE, Web of Science Core Collection and Embase are given in supplementary table 2.

This narrative critical review incorporates a structured literature search but was not registered with PROSPERO and is not presented as a PRISMA systematic review. The evidence is sparse and heterogeneous, particularly because no identified study simultaneously evaluated H_2, CKD, and a skeletal endpoint; formal quantitative synthesis would therefore be inappropriate. Mechanistic statements were graded using three prespecified evidence tiers:

- **Established:** reproducibly demonstrated in ≥2 independent laboratories, or in a human study with an appropriate comparator.
- **Suggested:** demonstrated in a single study or model system, awaiting replication.
- **Hypothesised:** a mechanistic inference drawn by the present authors from adjacent evidence, explicitly labelled as such and not supported by direct experimental data.

Records were screened by title and abstract and subsequently assessed in full text. Preclinical studies were considered against key SYRCLE domains, including randomisation, blinding, sample-size justification, and outcome completeness. Clinical studies were assessed for study design, comparator adequacy, blinding, confounding control, and funding or conflicts of interest. Because the clinical H_2 literature is concentrated within a limited number of research programmes, disclosures were considered when interpreting human findings.

Supplementary Table 1. Scope of the review defined by a PICOTS framework with inclusion and exclusion criteria.

Domain	Specification
**P — Population**	Adults with CKD stages G3a–G5; patients on maintenance haemodialysis or peritoneal dialysis; kidney transplant recipients with residual CKD-MBD. Comparator populations (post-menopausal osteoporosis, glucocorticoid-induced osteoporosis, disuse and diabetic bone loss) included only for mechanistic extrapolation, explicitly labelled as indirect evidence. Excluded: paediatric CKD; primary osteoporosis without CKD as a stand-alone topic.
**I — Intervention**	Molecular hydrogen (H_2) by any route: H_2-enriched dialysate (E-HD); inhaled H_2 gas or H_2–O_2 mixtures; oral hydrogen-rich water (HRW); hydrogen-rich saline (HRS); oral solid hydrogen capsules (OSHC), including hydrogen-rich coral calcium (HRCC). Excluded: hydrogen sulfide (H_2S), hydrogen peroxide, and "alkaline water" products where dissolved H_2 was not quantified.
**C — Comparator**	Conventional dialysate; placebo or sham; standard CKD-MBD care; untreated disease model controls; other antioxidant interventions (N-acetylcysteine, vitamin E, bardoxolone methyl, MitoQ) where used as mechanistic benchmarks.
**O — Outcomes**	*Primary skeletal outcomes: bone turnover markers (BSAP, intact PTH, TRAP-5b, P1NP, CTX); sclerostin, FGF23, soluble α-Klotho; areal and volumetric BMD (DXA, HR-pQCT); bone histomorphometry (turnover, mineralisation, volume); and fragility fracture. Secondary outcomes: systemic oxidative-stress/inflammatory indices, vascular calcification, and patient-reported symptoms. Mechanistic outcomes: ROS flux and redox-sensitive NRF2/KEAP1, NF-κB, FoxO, Wnt/β-catenin, HIF, NFATc1, RANKL/OPG, RUNX2, ALP, and cathepsin K pathways.*
**T — Time frame**	January 2005 to most recent indexing date, with priority to 2015–present. Landmark earlier studies defining bone redox biology and CKD-MBD pathophysiology retained without date restriction.
**S — Study designs**	Included: in vitro mechanistic studies in bone-cell systems; in vivo animal models; human observational and interventional studies; systematic reviews, meta-analyses and guideline documents. Excluded: case reports; abstracts without peer-reviewed full text; non-English without translation; commercial and non-peer-reviewed sources (used, where noted, only for study identification, never as evidence).
**Evidence grading**	Every mechanistic claim assigned as **Established** (≥2 independent replications or human comparator study), **Suggested** (single study/model), or **Hypothesised** (authors' inference; no direct data).

Supplementary Table 2. Database-specific Boolean search strategies.

Database	Concept block	Search string
**PubMed / MEDLINE**	A — Intervention	("molecular hydrogen"[tiab] OR "hydrogen gas"[tiab] OR "hydrogen-rich water"[tiab] OR "hydrogen rich saline"[tiab] OR "hydrogen-enriched"[tiab] OR "electrolyzed reduced water"[tiab] OR "electrolyzed hydrogen water"[tiab] OR "hydrogen-rich coral calcium"[tiab] OR "solid hydrogen"[tiab] OR "H_2 inhalation"[tiab] OR "Hydrogen"[Mesh])
	B — Kidney	("Renal Insufficiency, Chronic"[Mesh] OR "chronic kidney disease"[tiab] OR CKD[tiab] OR "end-stage renal"[tiab] OR "end stage kidney"[tiab] OR dialysis[tiab] OR h(a)emodialysis[tiab] OR "peritoneal dialysis"[tiab] OR uremi*[tiab] OR uraemi*[tiab])
	C — Bone	("Chronic Kidney Disease-Mineral and Bone Disorder"[Mesh] OR "renal osteodystrophy"[tiab] OR "CKD-MBD"[tiab] OR "Bone Diseases, Metabolic"[Mesh] OR osteoblast*[tiab] OR osteoclast*[tiab] OR osteocyte*[tiab] OR "bone remodeling"[tiab] OR "bone turnover"[tiab] OR sclerostin[tiab] OR RANKL[tiab] OR osteoprotegerin[tiab] OR "bone mineral density"[tiab] OR fracture*[tiab] OR "adynamic bone"[tiab])
	D — Redox	("Oxidative Stress"[Mesh] OR "oxidative stress"[tiab] OR "redox signal*"[tiab] OR "reactive oxygen species"[tiab] OR NRF2[tiab] OR NFE2L2[tiab] OR KEAP1[tiab] OR "NF-kappaB"[tiab] OR FOXO[tiab] OR "NADPH oxidase"[tiab] OR "hypoxia-inducible factor"[tiab])
	Combinations	#A AND #B; #A AND #C; #A AND #B AND #C; #A AND #C AND #D; #B AND #C AND #D. Filters: Humans OR Animals; English; 2005/01/01–present (Blocks A/B/C/D landmark searches undated).
**Web of Science Core Collection**	Combined	TS=("molecular hydrogen" OR "hydrogen-rich water" OR "hydrogen rich saline" OR "electrolyzed hydrogen water" OR "hydrogen-rich coral calcium" OR "hydrogen inhalation") AND TS=("chronic kidney disease" OR dialysis OR uremi* OR uraemi* OR "renal osteodystrophy" OR "CKD-MBD") AND TS=(bone OR osteoblast* OR osteoclast* OR osteocyte* OR fracture* OR "bone mineral density" OR sclerostin OR RANKL). Refined by Document Types = (Article OR Review); Languages = English; Timespan 2005–present. Citation-mapping performed on all included records.
**Embase (Elsevier)**	Combined	('hydrogen'/exp OR 'molecular hydrogen':ti,ab OR 'hydrogen rich water':ti,ab OR 'hydrogen rich saline':ti,ab OR 'electrolyzed water':ti,ab OR 'hydrogen rich coral calcium':ti,ab) AND ('chronic kidney failure'/exp OR 'hemodialysis'/exp OR 'peritoneal dialysis'/exp OR 'uremia'/exp OR ckd:ti,ab) AND ('bone'/exp OR 'renal osteodystrophy'/exp OR 'osteoblast'/exp OR 'osteoclast'/exp OR 'osteocyte'/exp OR 'bone density'/exp OR 'fracture'/exp OR sclerostin:ti,ab) AND [english]/lim AND [2005–current]/py. Emtree explosion enabled; conference abstracts excluded at screening.
**Trial registries**	ClinicalTrials.gov; EudraCT; WHO ICTRP	Condition: "chronic kidney disease" OR "kidney failure" OR "dialysis" OR "osteoporosis" OR "renal osteodystrophy"; Intervention: "hydrogen" OR "molecular hydrogen" OR "hydrogen water" OR "hydrogen rich coral calcium". All recruitment statuses.
**Supplementary methods**	—	Hand-search of reference lists of all included reviews; forward citation tracking of pivotal mechanistic papers; targeted author searches for the principal H_2–nephrology and H_2–bone research groups; grey-literature scan of institutional repositories (used for record identification only).
